# SLC25A20 promotes tumor growth by reprogramming energy metabolism in lung adenocarcinoma

**DOI:** 10.1038/s41419-026-09119-9

**Published:** 2026-07-23

**Authors:** Xing Liu, Xiaoyu Song, Cong Gan, Ye Wang, Mingxin Ni, Min Zhang, Shaodi Wen, Yahui Cao, Changhua Yi, Man Tian, Apeng Chen

**Affiliations:** 1 Department of Respiratory Medicine, Children’s Hospital of Nanjing Medical University, Nanjing, 210093 China; 2 The Second Hospital of Nanjing, Affiliated Hospital to Nanjing University of Chinese Medicine, Nanjing, 210003 China; 3 State Key Laboratory of Molecular Developmental Biology, Institute of Genetics and Developmental Biology, Chinese Academy of Sciences, 100101 Beijing, China; 4 Department of Oncology, The Affiliated Cancer Hospital of Nanjing Medical University & Jiangsu Cancer Hospital & Jiangsu Institute of Cancer Research, Nanjing, 210009 China; 5 Nanjing Key Laboratory of Pediatrics, Children’s Hospital of Nanjing Medical University, Nanjing, 210008 China

**Keywords:** Cancer metabolism, Cancer

## Abstract

Accumulating evidence has shown that the dysfunction of mitochondria, the multifunctional organelles in various cellular processes, is a pivotal event in the development of various diseases, including human cancers. The molecular alterations in tumors that promote metabolic diversity and distinct targetable dependencies remain poorly defined. The mitochondrial transporter SLC25A20, also known as carnitine-acylcarnitine translocase (CACT), is crucial for fatty acid β-oxidation (FAO) by importing acylcarnitines into the mitochondrial matrix. Here, we demonstrate that SLC25A20 is significantly upregulated in lung adenocarcinoma (LUAD) tissues and cells, and its expression correlates with poor patient survival. Gain- and loss-of-function experiments reveal that SLC25A20 promotes tumor cell proliferation, survival, migration, and invasion by enhancing FAO-associated mitochondrial bioenergetics and suppressing apoptosis. Mechanistically, SLC25A20-mediated FAO maintains mitochondrial function and supports oxidative phosphorylation (OXPHOS), thereby sustaining tumor bioenergetics and redox homeostasis. Pharmacological inhibition or genetic silencing of SLC25A20 impairs tumor growth in vivo. These findings establish SLC25A20 as a key metabolic driver in LUAD and suggest it may be a therapeutic target.

## Introduction

Lung adenocarcinoma (LUAD), the most prevalent histological subtype of non-small cell lung cancer (NSCLC), continues to pose a significant public health challenge worldwide [1–3]. Despite advances in targeted therapies and immunotherapies, the prognosis for patients with advanced LUAD remains poor, largely due to metastasis, therapy resistance, and tumor heterogeneity [4–7]. A hallmark of cancer is metabolic reprogramming, which enables tumor cells to sustain proliferative signaling, evade apoptosis, and adapt to nutrient stress [8, 9]. While the Warburg effect and glutamine addiction have long dominated the landscape of cancer metabolism, lipid metabolism, specifically the dynamic rewiring of fatty acid utilization, has emerged as a pivotal driver of cancer progression, particularly in LUAD [10–12].

Mitochondria are central organelles for cellular energy production and metabolic integration [13]. They orchestrate oxidative phosphorylation (OXPHOS), fatty acid oxidation (FAO), and reactive oxygen species (ROS) homeostasis [14–16]. The functional plasticity of mitochondrial metabolism is essential for oncogenesis and often correlates with dismal clinical outcomes [17, 18]. FAO, in particular, provides a vital source of ATP and NADPH, thereby fortifying tumor cells against metabolic stress and oxidative pressure [19–21]. Emerging evidence underscores a metabolic reliance of aggressive LUAD cells on FAO; however, the precise molecular “gatekeepers” governing mitochondrial fatty acid influx in this context remain largely elusive [22–25].

Mitochondrial FAO is strictly dependent on the carnitine shuttle system, a multi-step transport mechanism where Solute carrier family 25 member 20 (SLC25A20, also known as CACT) serves as the indispensable mediator for acylcarnitine/carnitine exchange across the inner mitochondrial membrane [26–30]. Despite the recognized importance of lipid rewiring in lung adenocarcinoma (LUAD) [31], and the burgeoning roles of SLC25 carriers in fueling malignancy [12, 32], SLC25A20 remains a remarkably overlooked frontier in oncology. While SLC25A20 has historically been studied in the context of carnitine-acylcarnitine translocase deficiency, its potential role in tumor metabolic adaptation remains poorly understood. Here, we hypothesized that SLC25A20 acts as a metabolic oncogene that orchestrates a specific lipid remodeling program to facilitate LUAD progression.

Here, we demonstrate that SLC25A20 is markedly overexpressed in LUAD tissues and correlates with poor patient prognosis. Through integrated transcriptomic, metabolomic, and functional analyses, we uncover a critical role of SLC25A20 in fueling FAO-driven bioenergetics and maintaining redox balance, which collectively suppress ROS-mediated apoptosis and empower tumor progression. These findings identify SLC25A20 as a novel metabolic vulnerability and a promising therapeutic target in lung adenocarcinoma.

## Materials and methods

### Cell culture

The cell lines used in this study included A549 (ATCC® CCL-185™), H1299 (ATCC® CRL-5803™), H1975 (ATCC® CRL-5908™), H1650 (ATCC® CRL-5883™), and BEAS-2B (ATCC® CRL-9609™). All cells were maintained in a humidified 5% CO₂ incubator at 37 °C. Culture media were supplemented with 10% FBS (Cellter Life, Cat. #FB2501), 1% penicillin/streptomycin (Proteinbio, Cat. #PC0315), and 2 mM L-glutamine (Gibco, Cat. #25030024).

The cells were authenticated by DNA fingerprinting and confirmed to be free from mycoplasma contamination (Microsynth, Bern, Switzerland). Viability was assessed using 0.1% trypan blue (Thermo Fisher Scientific, Cat. #15250061) and hemocytometer counts. Cryopreservation used serum-free cell cryopreservation medium (Proteinbio, Cat. #PAC1001041). All reagents were sourced from Gibco (media) and Proteinbio (FBS, trypsin, antibiotics) unless specified.

### Lung adenocarcinoma tissue sample collection

Tumor tissue specimens used in this study were obtained from Jiangsu Cancer Hospital (Nanjing, China). Written informed consent was obtained from all participants prior to the study, and all specimens were histologically confirmed as LUAD by independent pathologists. The investigation has been approved by the Ethics Committee of The Affiliated Cancer Hospital of Nanjing Medical University in Nanjing, China. The serial number is 2023K-Q078.

Sample Collection: A total of 28 patients with lung adenocarcinoma (LUAD) who underwent surgical resection at Jiangsu Cancer Hospital between January 2024 and March 2026 were enrolled (Supplementary Table 1). Strict inclusion criteria were applied: (i) histological confirmation of LUAD by two independent senior pathologists; and (ii) no history of neoadjuvant radiotherapy or chemotherapy prior to surgery. For each patient, matched pairs of tissues were harvested, including primary tumor tissues and adjacent noncancerous tissues (located at least 2 cm away from the tumor margin to ensure clear boundaries). A total of 28 matched sets of samples were successfully obtained. Upon resection, tissue specimens were rinsed with sterile saline, immediately flash-frozen in liquid nitrogen, and stored at −80 °C for subsequent Western blot and qPCR analyses.

Paired Analysis Design: To minimize the confounding effects of inter-individual genetic heterogeneity, a paired-sample design was implemented for all quantitative molecular analyses. Specifically, each tumor sample was directly compared with its autologous adjacent noncancerous counterpart from the same patient.

### Kaplan–Meier survival analysis

Survival analyses were performed using the Kaplan–Meier Plotter database (https://kmplot.com/analysis/). Patients were stratified into high- and low-expression groups based on the auto-select best cutoff option implemented in Kaplan–Meier Plotter. Overall survival (OS) and progression-free survival (PFS) were analyzed separately. Univariate Cox regression analysis was applied, and the “exclude biased arrays” option was enabled for array quality control, while all remaining parameters were maintained at their default settings.

#### In vivo animal assays

Eight-week-old male BALB/c nude mice were randomly assigned to the experimental groups. A single-cell suspension containing 2 × 10^6^ cells (SLC25A20-OE/NC or SLC25A20-KO/NC) in 100 μL of sterile PBS was subcutaneously injected into the flank of each mouse. Tumor growth kinetics were monitored every five days using digital calipers. At the endpoint, subcutaneous tumors were excised, photographed, weighed, and fixed in 4% paraformaldehyde for subsequent IHC analysis.

### Metabolomics

#### Sample preparation

The metabolites were extracted from the cell residue using 1 mL of precooled methanol/acetonitrile/water (v/v, 2:2:1) under sonication for 1 h in ice baths. The mixture was incubated at –20 °C for 1 h, followed by centrifugation at 16,000 × *g*, 4 °C for 20 min, and then transferred to a sampling vial for LC-MS analysis by Shanghai Bioprofile Technology Co., Ltd.

Additionally, to ensure data quality for metabolic profiling, quality control (QC) samples were prepared by pooling aliquots of all samples representative of the entire dataset and used for data normalization. QC samples were prepared and analyzed using the same procedure as the experimental samples in each batch. Dried extracts were then dissolved in 50% acetonitrile. Each sample was filtered through a disposable 0.22 µm cellulose acetate filter and transferred into 2 mL HPLC vials, then stored at −80 °C until analysis.

#### UHPLC-MS analysis

The LC/MS portion of the platform was based on a Shimadzu Nexera X2 LC-30AD system equipped with an Acquity UPLC HSS T3 column (1.8 μm 2.1 × 50mm Column, Waters) and a triple quadrupole mass spectrometer (5500 QTRAP, AB SCIEX). Metabolites were detected in electrospray negative-ionization and positive-ionization modes. A 5 μL samples were injected sequentially with the LC autosampler. The Acquity UPLC HSS T3 column (1.8 μm 2.1 × 50 mm Column, Waters) was heated to 40 °C under a flow rate of 200 μL/min. A gradient was used to separate the compounds, consisting of 0.1% formic acid aqueous solution (solvent A) and 100% acetonitrile (solvent B). The gradient started at 100% solvent A for 2.5 min and increased linearly to 70% solvent A over 9 min, and then increased linearly to 0% solvent A over 1 min, followed by a 5.4 min hold before returning to the starting mixture during 0.1 min and re-equilibrating for 2.5 min. QC samples were injected every several samples during acquisition.

The MS conditions were set as follows: negative-ionization: Source Temperature 550 °C, Ion Source Gas1 (GAS1): 40, Ion Source Gas2 (GAS2): 50, Curtain Gas (CUR): 35, Ion Spray Voltage Floating (ISVF): −4500 V; positive-ionization: Source Temperature 550 °C, Ion Source Gas1 (GAS1): 40, Ion Source Gas2 (GAS2): 50, Curtain Gas (CUR): 35, Ion Spray Voltage Floating (ISVF): 5500 V. Transitions were detected by MRM mode.

#### Data processing and statistical analysis

The raw MRM data were processed using MultiQuant 3.0.2 software to integrate peak areas. For multivariate analysis, orthogonal partial least squares discriminant analysis (OPLS-DA) was performed. Discriminatory metabolites were identified based on a variable importance in projection (VIP) value > 1.0 and a *p* value < 0.05 determined by two-tailed Student’s *t-*test or one-way ANOVA. Hierarchical clustering analysis was performed using the R package to visualize metabolic profiles.

### RNA sequencing and bioinformatics

#### Analysis RNA extraction and library construction

Total RNA was extracted and purified using TRIzol reagent (Invitrogen, Carlsbad, CA, USA) following the manufacturer’s protocol. The concentration and purity of the RNA were determined using a NanoDrop ND-1000 spectrophotometer (NanoDrop, Wilmington, DE, USA), and RNA integrity was assessed with an Agilent 2100 Bioanalyzer (Agilent, CA, USA); only samples with an RNA Integrity Number (RIN) > 7.0 were used for subsequent library preparation. Poly(A) RNA was isolated from 1 μg of total RNA using two rounds of purification with Dynabeads Oligo(dT) (Thermo Fisher, CA, USA). The purified mRNA was then fragmented using the Magnesium RNA Fragmentation Module (NEB, Ipswich, MA, USA) at 94 °C for 5–7 min. Subsequently, the RNA fragments were reverse-transcribed into cDNA using SuperScript™ II Reverse Transcriptase (Invitrogen, USA). Second-strand cDNA synthesis was performed using E. coli DNA polymerase I (NEB), RNase H (NEB), and dUTP solution (Thermo Fisher) to generate strand-specific libraries. An A-base was added to the blunt ends of the cDNA fragments for ligation to indexed adapters. Following adapter ligation and size selection with AMPure XP beads (Beckman Coulter), the U-labeled second strands were degraded using the heat-labile UDG enzyme (NEB). The remaining cDNA products were amplified via PCR under the following conditions: 95 °C for 3 min; 8 cycles of 98 °C for 15 s, 60 °C for 15 s, and 72 °C for 30 s; and a final extension at 72 °C for 5 min. The final libraries had an average insert size of 300 ± 50 bp. Finally, paired-end sequencing was performed on an Illumina NovaSeq 6000 platform using a 2 × 150 bp read length by LC-Bio Technology Co., Ltd. (Hangzhou, China).

#### Bioinformatics analysis of RNA-seq

The fastp software (https://github.com/OpenGene/fastp) was used to remove reads containing adaptor contamination, low-quality bases, and undetermined bases, using the default parameters. The sequence quality was also verified using fastp. We used HISAT2 (https://ccb.jhu.edu/software/hisat2) to map reads to the human reference genome (GRCh38). The mapped reads from each sample were assembled using StringTie (https://ccb.jhu.edu/software/stringtie) under default parameters. Subsequently, transcriptomes from all samples were merged using gffcompare (https://github.com/gpertea/gffcompare/) to reconstruct a comprehensive transcriptome.

After generating the final transcriptome, StringTie was employed to estimate the expression levels of mRNAs by calculating Fragments Per Kilobase of transcript per Million mapped reads (FPKM) using the following formula: FPKM (FPKM = [total exon fragments /mapped reads(millions) × exon length(kb)]). The differentially expressed mRNAs were selected with fold change >2 or fold change <0.5 and with parametric F-test comparing nested linear models (*p* value < 0.05) by the R package edgeR (https://bioconductor.org/packages/release/bioc/html/edgeR.html).

#### Immunofluorescence staining and confocal imaging

For immunofluorescence analysis, cultured cells grown on coverslips or tissue sections were fixed, permeabilized, blocked, and incubated with the indicated primary antibodies, including anti-E-cadherin (Proteintech, 20874-1-AP, 1:1,000), anti-Vimentin (Proteintech, 10366-1-AP, 1:1,000), anti-Cleaved caspase-3 (Proteintech, 87055-4-RR, 1:1,000). Following three washes with PBS-Tween-20, the sections were incubated with corresponding fluorophore-conjugated secondary antibodies for 1 h at room temperature in the dark. Nuclei were counterstained with DAPI.

High-resolution images were captured using a Leica TCS SP8 confocal microscope and a Nikon Ti-E&N-STORM super-resolution microscope system. For quantification, the number of positive cells or the fluorescence intensity was calculated from at least three random fields per section using ImageJ software.

#### Quantitative real-time PCR (qRT-PCR)

Total RNA was extracted from LUAD cells using an RNA purification kit (Qiagen, Germany) and reverse-transcribed into cDNA using the PrimeScript RT Reagent Kit (Takara, Japan) according to the manufacturer’s instructions. qRT-PCR was performed using specific primers (listed in Supplementary Table 3). β-actin was used as an endogenous control, and the relative expression of the target genes was calculated using the 2^^-ΔΔCt^ method.

#### Protein extraction and Western blot analysis

Cells were lysed in RIPA buffer (50 mM Tris pH 7.4, 150 mM NaCl, 1% NP-40, 0.5% sodium deoxycholate, 0.1% SDS) supplemented with a protease and phosphatase inhibitor cocktail. Protein concentrations were quantified using the BCA Protein Assay Kit (Thermo Scientific, UK). Approximately 40 μg of total protein per sample was separated by SDS-PAGE and transferred onto PVDF membranes. The membranes were blocked with 5% non-fat milk at room temperature for 1 h and then incubated overnight at 4 °C with the following primary antibodies: anti-SLC25A20 (Proteintech, 15170-1-AP, 1:1,000), anti-FASN (HUABIO, ET1701-91, 1:2,000), anti-HMOX1 (HUABIO, HA721854, 1:2,000), anti-E-cadherin (Proteintech, 20874-1-AP, 1:1,000), anti-Vimentin (Proteintech, 10366-1-AP, 1:1,000), anti-pro-Caspase-3 (HUABIO, ET1608-64, 1:1,000), anti- Cleaved caspase-3 (Proteintech, 87055-4-RR, 1:1,000), anti-β-actin (Sigma, A5441, 1:5000), anti-GAPDH (Proteintech, 10494-1-AP, 1:5000).

#### Wound healing assays

Cells were seeded in 6-well plates and cultured until they reached approximately 90–100% confluency. A linear wound was created by scratching the cell monolayer with a sterile 200 μL pipette tip. After washing with PBS to remove cellular debris, the cells were cultured in medium supplemented with 1% FBS. Images of the wounds were captured at 0 and 24 h using an inverted microscope. The wound closure area was quantified using ImageJ software.

#### Cell migration and invasion assays

Migration and invasion assays were conducted using Transwell chambers with 8 μm pores. For the invasion assay, the upper chamber was pre-coated with Matrigel. Cells were seeded into the upper chamber in serum-free medium, while medium containing 20% FBS was added to the lower chamber as a chemoattractant. After incubation for 18–30 h, cells that migrated or invaded to the lower membrane surface were fixed with 4% PFA, stained with 0.1% crystal violet, and photographed using an inverted microscope.

#### Cell counting and growth curve analysis

Cells were seeded into 6-well plates at an initial density of 2 × 10^4^ cells per well. Over a consecutive 7-day period, cells from triplicate wells were trypsinized and resuspended every 24 h. Cell counts were performed using an automated cell counter or a hemocytometer with Trypan blue (0.4%) exclusion to ensure that only viable cells were quantified. The growth curve was generated by plotting the total number of viable cells against the respective time points.

#### CCK-8 cell proliferation and colony formation assays

For the CCK-8 cell proliferation assay, lung adenocarcinoma cells (2,000 cells/well) were seeded into 96-well plates. After incubation for 12 h, 10 μL of Cell Counting Kit-8 (CCK-8) reagent was added to each well and incubated for 1.5 h at 37 °C. Cell viability was subsequently measured at 450 nm using a microplate reader. Measurements were performed at the indicated time points to evaluate cell proliferation.

For the colony formation assay, 500 cells were seeded into 6-well plates and cultured for 14 days. Colonies were stained with crystal violet (0.5% dissolved in 25% ethanol). The number of colonies was counted using ImageJ software. In detail, first, ROIs were set according to the boundaries of the wells on the plate, and the outside area was removed. Second, the original images were converted to 8-bit grayscale, and then the brightness/contrast were adjusted to identify the edges of the colonies. Third, a threshold was set to separate the colonies well from the background. Next, the images were binary processed to fill holes, perform a watershed to divide contiguous clones, and then analyze the particles. Determine the colony by setting the size and circularity as the following parameters: size: 100-infinity, circularity: 0.2–1.

#### Flow cytometry analysis of cell cycle and apoptosis

Flow Cytometry Analysis of cell cycle and apoptosis cell cycle distribution was analyzed using a cell-cycle analysis kit (C-6031, US Everbright Inc). Briefly, LUAD cells with varying SLC25A20 expression levels were harvested and fixed in 80% cold ethanol at -20 °C for at least 2 h. After washing, the cells were incubated with 0.5 mL of propidium iodide (PI) staining solution for 20 min in the dark at 4 °C. The cell-cycle distribution was then evaluated using a flow cytometer (Beckman Coulter, Fullerton, CA).

Cell apoptosis was determined using an Annexin V-FITC/PI Apoptosis Detection Kit (F-6012, US Everbright Inc). Briefly, cells were collected, resuspended in binding buffer, and stained with 5 μL of FITC-conjugated Annexin V and 5 μL of PI for 15 min at room temperature in the dark. The apoptotic rate was subsequently analyzed by flow cytometry (Beckman Coulter, Fullerton, CA).

#### Measurement of oxygen consumption rates

The oxygen consumption rate (OCR) was determined using a Seahorse XF96 Extracellular Flux Analyzer.

Cells were plated onto Seahorse XF-96 cell culture microplates at a density of 30,000 cells per well in Dulbecco’s Modified Eagle Medium (DMEM) enriched with 10% fetal bovine serum (FBS). After 12 h of incubation, the culture medium was replaced with Seahorse XF Base Medium (pH 7.4), supplemented with 10 mM glucose, 1 mM pyruvate, and 2 mM glutamine. Cells were then incubated in a non-CO_2_ incubator at 37 °C for 1 h to allow for pH and temperature equilibration. The basal OCR was recorded for three cycles and normalized to the total protein concentration of each well, as determined by a BCA assay. Data analysis was performed using Wave software (Agilent Technologies).

#### Statistical analysis and data visualization

Statistical analyses and data visualization were performed using the R statistical computing environment (version 4.3.0). Principal component analysis (PCA) was executed via the prcomp function using singular value decomposition, and visualization was facilitated by the factoextra package. To assess time-dependent variations in lipid abundance, one-way analysis of variance (ANOVA) was employed, followed by Tukey’s Honestly Significant Difference (HSD) post hoc tests for multiple comparisons. Results were presented as bar charts, where different letters denote statistically significant differences (*P* < 0.05), while shared letters indicate no significant difference (*P* > 0.05). Hierarchical clustering and heatmap generation were conducted using the pheatmap package.

#### Measurement of intracellular ATP levels

Intracellular ATP concentrations were determined using an ATP Assay Kit (Servicebio, G4309-96T) based on a firefly luciferase system. Briefly, cells were harvested and lysed with an ATP-releasing buffer on ice. After centrifugation at 12,000 × *g* for 5 min, the supernatant was collected and mixed with the ATP detection working solution. The luminescence was measured using a microplate reader. ATP levels were normalized to viable cell number.

#### Generation of SLC25A20 knockout cell lines

SLC25A20 knockout (KO) LUAD cell lines were generated using the CRISPR/Cas9 system. Briefly, specific small-guide RNAs (sgRNAs) targeting the human SLC25A20 genomic locus were designed and cloned into the pLentiCRISPRv2 vector. Cells were transfected with the recombinant plasmids using Lipofectamine 3000, followed by selection with puromycin to establish stable pools. The sequences of the sgRNAs are provided in Supplementary Table 4.

#### Generation of SLC25A20 overexpression cell lines

To generate SLC25A20 overexpression (OE) cell lines, the full-length human SLC25A20 cDNA was cloned into the pLenti-CMV-MCS-mCherry lentiviral vector. Lentiviral particles were produced by co-transfecting HEK-293T cells with the recombinant plasmid and packaging plasmids (psPAX2 and pMD2.G) using Lipofectamine 3000. LUAD cells were then infected with the viral supernatant. Stable cell lines were established and identified by mCherry fluorescence visualization and Western blotting. Cells infected with the empty mCherry vector served as the negative control.

## Results

### SLC25A20 is upregulated in LUAD and associated with unfavorable clinical outcomes

**Fig. 1:**
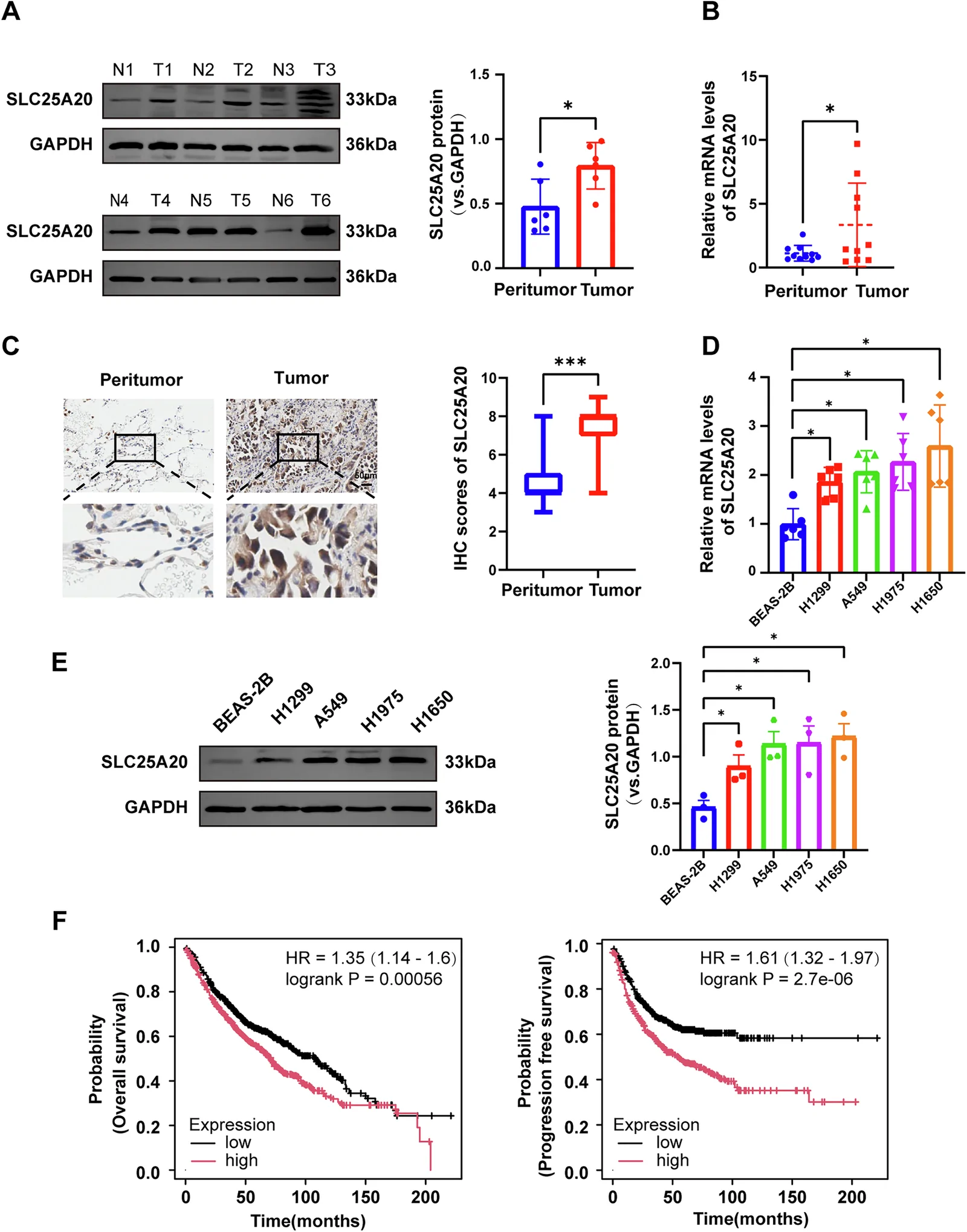
SLC25A20 is upregulated in LUAD and is associated with poor prognosis. **A** Representative Western blot analysis and quantification of SLC25A20 protein expression in paired LUAD tumor tissues and adjacent peritumor tissues. GAPDH was used as the loading control. N, matched adjacent non-tumor tissue; T, matched tumor tissue. **B** qRT-PCR analysis of *SLC25A20* mRNA expression in paired LUAD tumor tissues and adjacent peritumor tissues. **C** Representative IHC staining of SLC25A20 in paired LUAD tumor tissues and adjacent peritumor tissues. Representative enlarged images and quantification of IHC scores are shown. Scale bar: 50 μm. **D** qRT-PCR analysis of SLC25A20 mRNA expression in BEAS-2B cells and LUAD cell lines. **E** Western blot analysis and quantification of SLC25A20 protein expression in BEAS-2B cells and LUAD cell lines. GAPDH was used as the loading control. **F** Kaplan–Meier analysis of overall survival and progression-free survival in LUAD patients stratified by SLC25A20 expression. The association between SLC25A20 expression and clinical outcomes, including overall survival (OS, left) and progression-free survival (PFS, right), was analyzed using the KM-Plotter database. The specific parameters: Probe ID: 203658_at; patients were stratified into high and low expression groups using the “Auto-select best cutoff” algorithm; and the analysis was restricted to the “adenocarcinoma” histological subtype (*n* = 672 for OS; *n* = 443 for PFS). Hazard ratios (HRs) with 95% confidence intervals and log-rank *P* values are indicated. Data are presented as mean ± SD. Statistical significance was determined by paired two-tailed Student’s *t*-test for paired tumor and peritumor tissue comparisons, unpaired two-tailed Student’s *t* test or one-way ANOVA for cell-line comparisons, and log-rank test for survival analyses. *P < 0.05, ***P < 0.001.

To assess the expression of SLC25A20 in LUAD, we examined its levels in adjacent non-tumor and LUAD tissues utilizing Western blot and qPCR assays (Fig. 1A, B). Consistently, immunohistochemistry (IHC) staining in an independent LUAD cohort revealed significantly elevated SLC25A20 protein levels in tumor tissues compared to matched non-tumor tissues (Fig. 1C). Although SLC25A20 is documented as a mitochondrially localized protein in the MitoCarta 3.0 database, its specific distribution in LUAD cells had not been characterized. Through immunofluorescence (IF) co-staining of SLC25A20 and mitochondria in A549 cells and U2OS cells, a human osteosarcoma cell line commonly used for subcellular localization imaging, we confirmed that SLC25A20 is localized to the mitochondria (Fig. S1A, B).

Additionally, SLC25A20 expression was higher in a series of human LUAD cell lines (H1299, A549, H1975, and H1650) compared to the human normal bronchial epithelial cell line (BEAS-2B) (Fig. 1D, E). Moreover, survival analysis of public gene chip expression datasets using the online Kaplan–Meier Plotter [33] tool indicated that upregulation of SLC25A20 was associated with shorter overall survival and progression-free survival in LUAD patients (Fig. 1F). Notably, additional analyses using TCGA-LUAD and GEPIA2 datasets suggested that the prognostic significance of SLC25A20 is dependent on molecular context. While no significant association was observed in the overall TCGA-LUAD cohort, SLC25A20 expression was significantly associated with survival outcomes in specific LUAD molecular subtypes, including the TRU and PP subtypes (Fig. S1C-E). Together, these findings indicate that SLC25A20 is upregulated in LUAD and associated with unfavorable clinical outcomes.

### SLC25A20 promotes LUAD cell growth in vitro

**Fig. 2:**
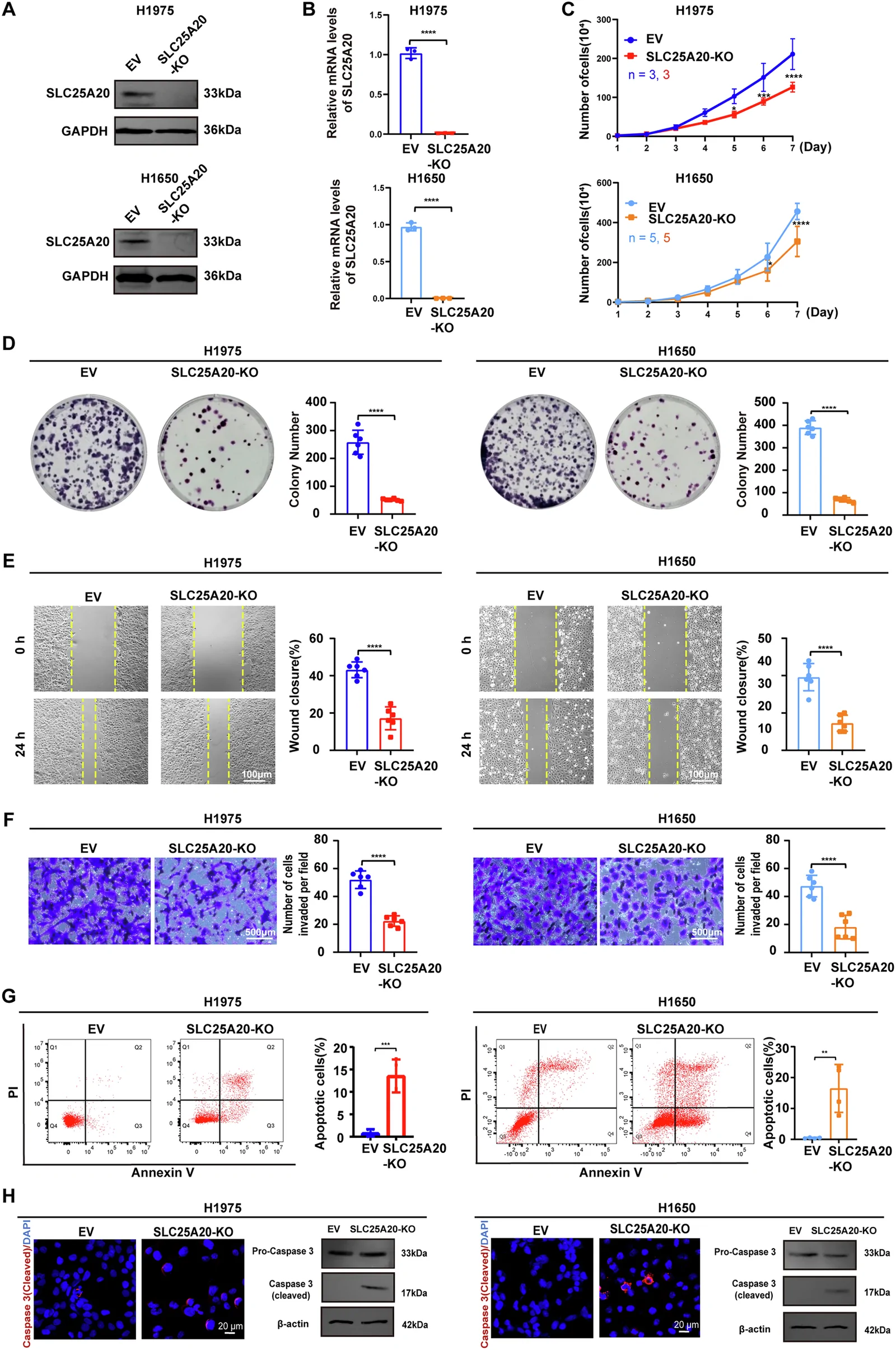
*SLC25A20* knockout suppresses LUAD cell proliferation, migration, and invasion and promotes apoptosis. **A**, **B** Validation of *SLC25A20* knockout in H1975 and H1650 cells by Western blotting and qRT-PCR analysis. GAPDH was used as the loading control for Western blotting. **C** Cell growth curves of EV and *SLC25A20*-KO H1975 and H1650, as determined by direct viable cell counting at the indicated time points. **D** Colony formation assays of EV and SLC25A20-KO H1975 and H1650 cells. Representative images of colonies and quantification of colony numbers are shown. **E** Wound-healing assays showing the migratory ability of EV and *SLC25A20*-KO H1975 and H1650 cells. Representative images obtained at 0 h and 24 h and quantification of wound closure are shown. Scale bar: 100 μm. **F** Transwell invasion assays showing the invasive ability of EV and *SLC25A20*-KO H1975 and H1650 cells. Representative images and quantification of invaded cells per field are shown. Scale bar: 500 μm. **G** Representative flow cytometry plots and quantification of apoptotic cells in EV and *SLC25A20*-KO H1975 and H1650 cells, as determined by Annexin V/PI staining. Apoptotic cells were defined as early apoptotic cells. **H** Representative IF and Western blot analysis of cleaved caspase-3 in EV and SLC25A20-KO H1975 and H1650 cells. Nuclei were counterstained with DAPI. β-actin was used as the loading control for Western blotting. Scale bar: 20 μm. Data are presented as mean ± SD from three independent experiments. Statistical significance was determined by two-tailed Student’s *t* test for two-group comparisons and two-way ANOVA for cell growth curves. **P < 0.01, ***P < 0.001.

To investigate the role of SLC25A20 in LUAD progression, we generated *SLC25A20* knockout (KO) cell lines using the CRISPR/Cas9 system to abolish SLC25A20 expression in H1975 and H1650 cells (Fig. S1F, H). Western blot and qRT-PCR analysis confirmed that SLC25A20 protein and mRNA levels were efficiently eliminated (Fig. 2A, B). Functional assays revealed that SLC25A20 knockout markedly suppressed the proliferation and colony formation of H1975 and H1650 cells (Fig. 2C, D; Fig. S2A, B). Furthermore, the cell migration and invasion abilities of these cells were significantly impaired upon SLC25A20 depletion (Fig. 2E, F).

Considering that suppressed LUAD growth may stem from cell cycle arrest and/or increased apoptosis, we subsequently evaluated the effect of SLC25A20 knockout on cell cycle distribution and apoptosis using flow cytometry. Strikingly, knockout of SLC25A20 significantly induced G2/M phase arrest and triggered apoptosis in H1975 and H1650 cells (Fig. S2C, D). Additionally, increased cleaved-caspase-3 expression further supported the conclusion that SLC25A20 deletion leads to programmed cell death (Fig. 2G, H). Collectively, these data suggest that SLC25A20 promotes proliferation and inhibits cell apoptosis in LUAD cells in vitro.

### Forced expression of SLC25A20 promotes LUAD cell invasion and migration and alters selected EMT-related markers

**Fig. 3:**
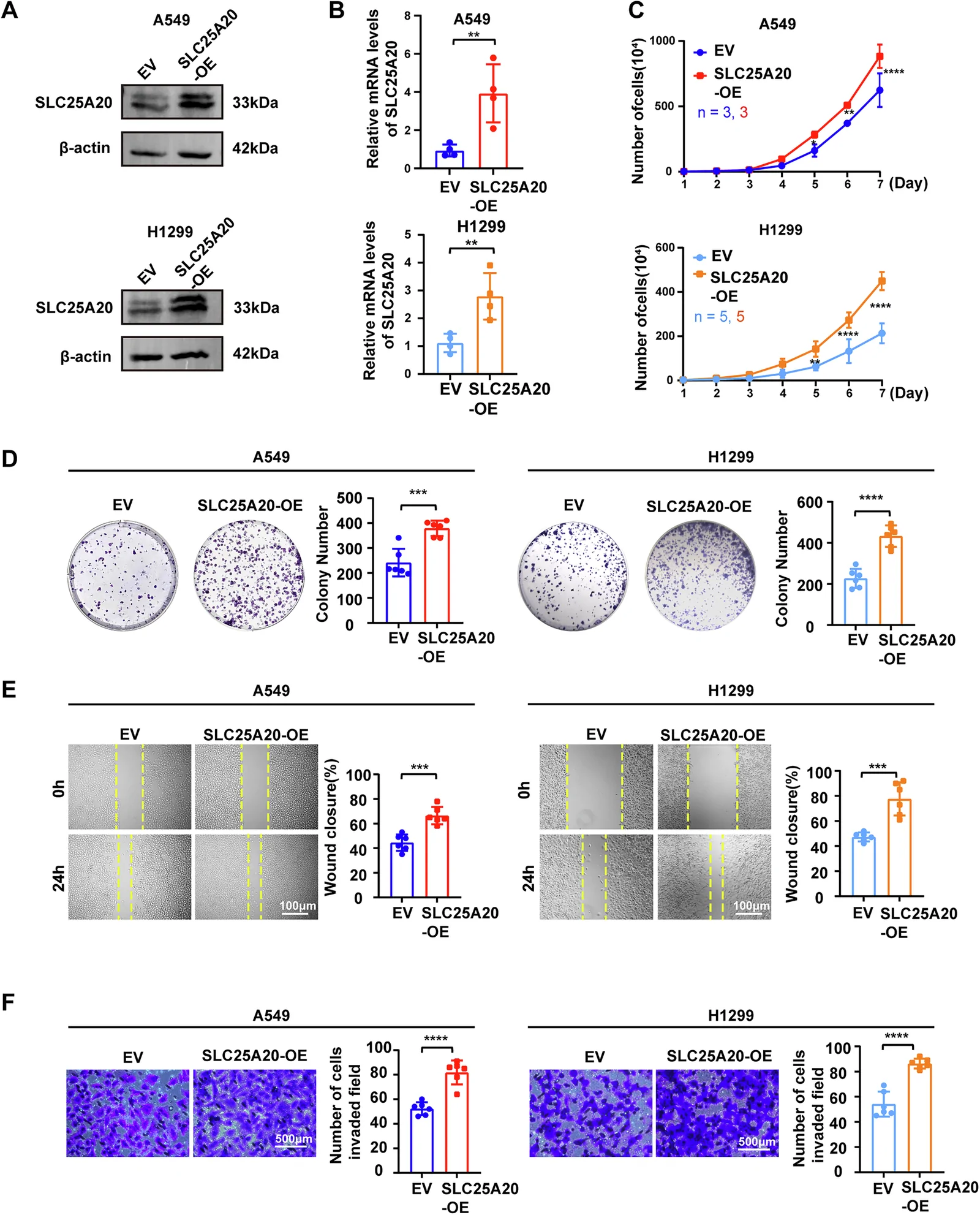
SLC25A20 promotes LUAD cell proliferation, migration and invasion. **A**, **B** Western blotting and qRT-PCR validation of SLC25A20 overexpression in A549 and H1299 cells. β-actin served as the loading control. **C** Growth curves of EV and SLC25A20-overexpressing A549 and H1299 cells assessed by direct viable cell counting. **D** Representative colony formation images and quantification of colony numbers in EV and SLC25A20-overexpressing A549 and H1299 cells. **E** Representative wound-healing images and quantification of wound closure in EV and SLC25A20-overexpressing A549 and H1299 cells. Scale bar: 100 μm. **F** Representative Transwell invasion images and quantification of invaded cells in EV and SLC25A20-overexpressing A549 and H1299 cells. Scale bar: 500 μm. Data are shown as mean ± SD from three independent experiments. Statistical significance was assessed using two-tailed Student’s *t*-test for two-group comparisons and two-way ANOVA for growth curves. **P < 0.01, ***P < 0.001, ****P < 0.0001.

Next, we overexpressed SLC25A20 in A549 and H1299 cells by transfecting the cells with the constructed SLC25A20 overexpression plasmid (Fig. 3A, B; Fig. S1G, I). SLC25A20 overexpression in A549 and H1299 cells resulted in a significant increase in cell proliferation and colony formation compared with the control group (Fig. 3C, D; Fig. S2E, F), while cell migration and invasion abilities were also enhanced (Fig. 3E, F). Given that EMT-related phenotypic changes are closely associated with cancer cell migration and invasion, the effect of SLC25A20 overexpression on the expression of selected EMT-associated markers was evaluated by immunofluorescence staining and Western blotting (Fig. S3A, B). SLC25A20 overexpression was associated with increased Vimentin expression and decreased E-cadherin expression.

These results suggest that SLC25A20 overexpression promotes LUAD cell migration and invasion and is accompanied by EMT-like marker changes, including increased Vimentin expression and decreased E-cadherin expression.

### SLC25A20 promotes lung tumor growth in vivo

**Fig. 4:**
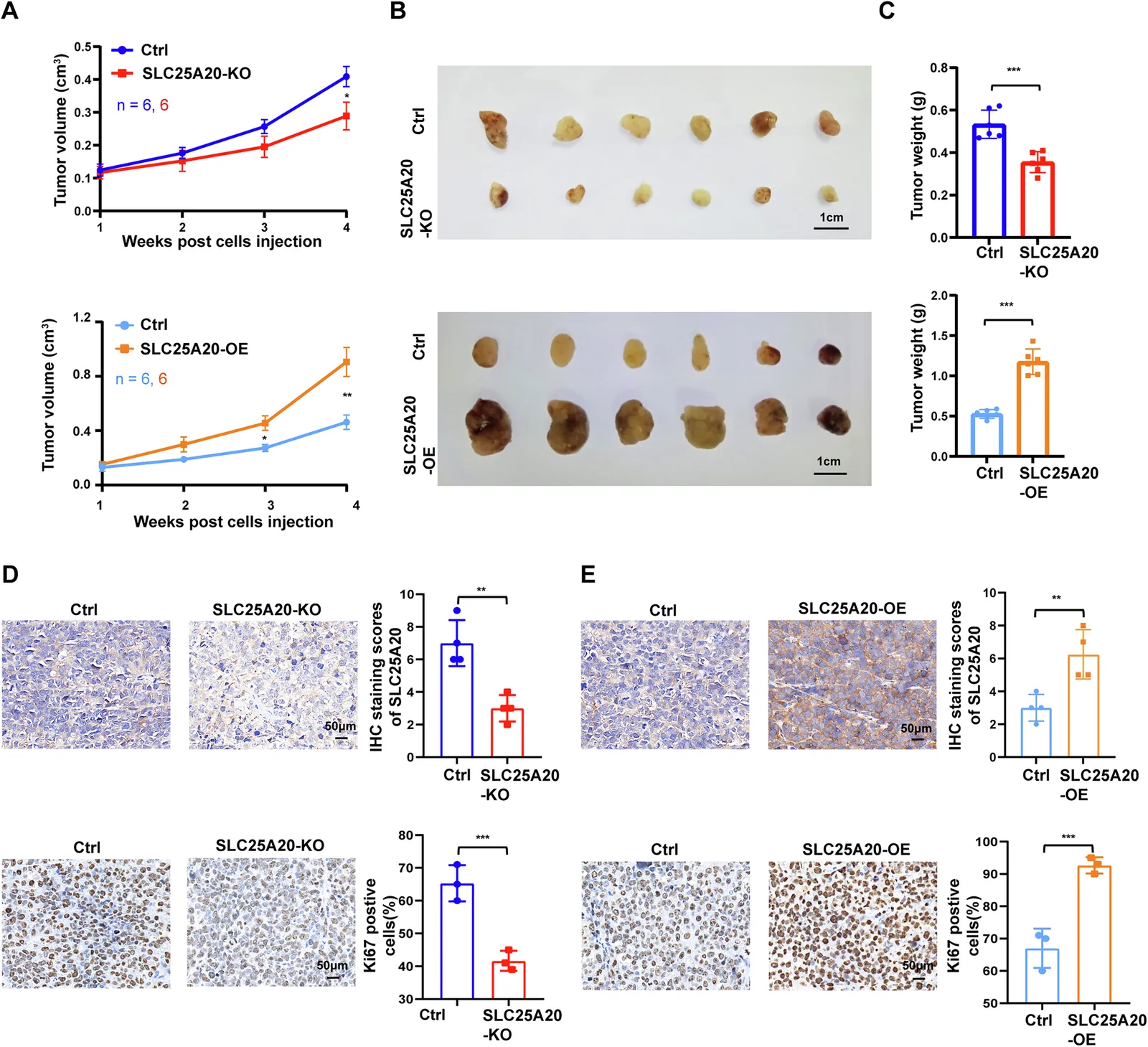
SLC25A20 promotes LUAD xenograft tumor growth in vivo. **A** Tumor growth curves of subcutaneous xenografts derived from control and *SLC25A20*-KO cells, and from control and SLC25A20-OE cells. Tumor volumes were measured weekly after cell injection. *n* = 6. **B** Representative images of excised subcutaneous xenograft tumors from the indicated groups. Scale bar: 1 cm. *n* = 6. **C** Endpoint tumor weights of xenografts from the indicated groups. *n* = 6. **D** Representative immunohistochemical staining and quantification of SLC25A20 and Ki67 in xenograft tumors derived from control and *SLC25A2*0-KO cells. Scale bar: 50 μm. **E** Representative IHC and quantification of SLC25A20 and Ki67 in xenograft tumors derived from control and SLC25A20-OE cells. Scale bar: 50 μm. Data are presented as mean ± SD. Statistical significance was determined by two-way ANOVA for tumor growth curves and two-tailed Student’s *t*-test for two-group comparisons. *P < 0.05, **P < 0.01, ***P < 0.001.

To clarify the in vivo biological functions of SLC25A20, we established subcutaneous xenograft tumor models in BALB/c nude mice. Tumor growth kinetics and weights at the endpoint showed that the growth of LUAD was significantly inhibited by *SLC25A20* knockout in H1975 cells (Fig. 4A–C). In contrast, tumor progression was promoted by SLC25A20 overexpression in A549 cells. IHC analysis of SLC25A20 and the proliferation marker Ki67 indicated that *SLC25A20* knockout significantly reduced the percentage of Ki67-positive cells (Fig. 4D). Conversely, SLC25A20 overexpression resulted in a significant elevation in proliferative activity (Fig. 4E). Collectively, these data indicate that SLC25A20 deficiency suppresses the in vivo growth of lung tumors, further supporting its role as an oncogene in LUAD progression.

### SLC25A20 remodels lipid metabolic and mitochondrial bioenergetic transcriptional programs in LUAD cells

**Fig. 5:**
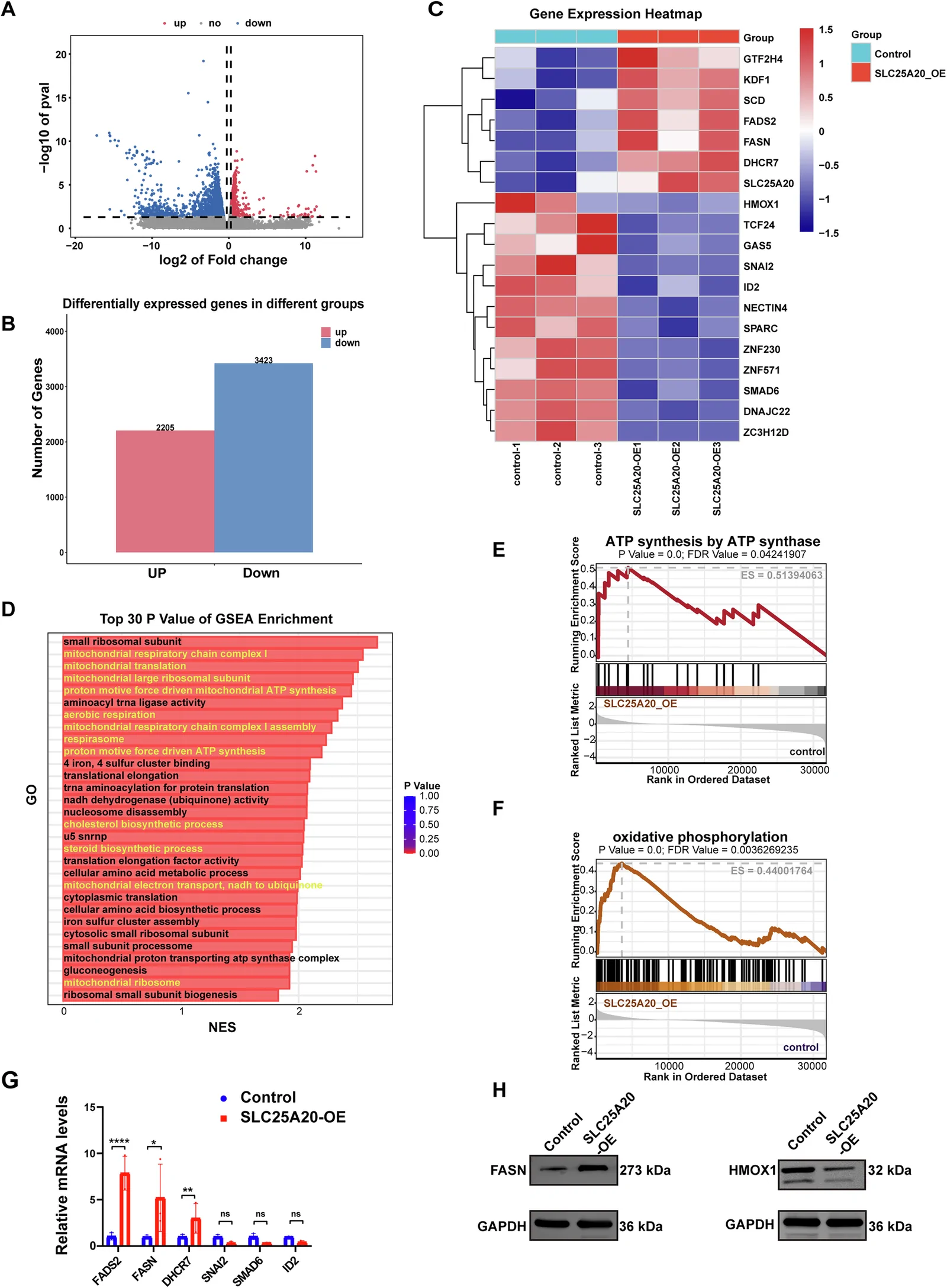
SLC25A20 reprograms lipid metabolism and mitochondrial bioenergetic pathways in LUAD cells. **A** Volcano plot showing differentially expressed genes between SLC25A20-overexpressing and control A549 cells based on RNA-seq analysis. Red and blue dots indicate significantly upregulated and downregulated genes, respectively. **B** Number of significantly upregulated and downregulated genes identified in SLC25A20-overexpressing A549 cells compared with control cells. **C** Heatmap showing representative differentially expressed genes in control and SLC25A20-overexpressing A549 cells. **D** Gene Ontology biological process enrichment analysis of differentially expressed genes in SLC25A20-overexpressing A549 cells. The top 30 enriched biological processes are shown. **E**, **F** Gene set enrichment analysis showing enrichment of ATP synthesis by ATP synthase and oxidative phosphorylation gene sets in SLC25A20-overexpressing A549 cells. **G** qRT-PCR validation of selected differentially expressed genes in control and SLC25A20-overexpressing A549 cells. **H** Western blotting of FASN and HMOX1 protein expression in control and SLC25A20-overexpressing A549 cells. GAPDH was used as the loading control. Data are presented as mean ± SD from three independent experiments where applicable. Statistical significance was determined by two-tailed Student’s *t-*test. *P < 0.05, **P < 0.01.

To further explore the molecular mechanism by which SLC25A20 regulates LUAD cell proliferation, we performed RNA-seq analysis in control A549 cells and A549 cells overexpressing SLC25A20. Analysis of the RNA-seq data via a volcano plot highlighted a range of significant transcriptional shifts, including several key genes subsequently found to be involved in metabolic regulation (Fig. 5A, B). Heatmap results demonstrated that, SLC25A20 overexpression enhanced the expression of FADS2, FASN, and SCD at the transcriptional level (Fig. 5C). Together, these findings suggest that SLC25A20 enhances the fatty acid synthesis pathway. The expression pattern was validated at multiple levels as follows: (i) Functional enrichment analysis of the DEGs underscored a significant shift in metabolic and structural pathways. Notably, GSEA and GO enrichment highlighted the activation of cholesterol and steroid biosynthetic processes, alongside a robust enrichment in mitochondrial functions, including the respiratory chain (Complex I) and mitochondrial translation (Fig. 5D; Fig. S4C–H). (ii) Gene Set Enrichment Analysis (GSEA) further confirmed that SLC25A20 overexpression significantly upregulated pathways associated with energy metabolism, specifically oxidative phosphorylation and ATP synthesis by ATP synthase (Fig. 5E, F). (iii) Transcriptional profiling via heatmap analysis revealed that SLC25A20 upregulation was associated with coordinated increases in key lipogenic enzymes, including FASN, FADS2, SCD, and DHCR7 (Fig. 5C). Consistent with the transcriptomic data, qRT-PCR analysis further confirmed the upregulation of FASN, FADS2, and DHCR7 (Fig. 5G). Western blotting further showed increased FASN and decreased HMOX1 protein expression in SLC25A20-overexpressing cells (Fig. 5H), consistent with SLC25A20-associated lipid metabolic remodeling and altered oxidative-stress–responsive signaling. (iv) Intriguingly, the heatmap also displayed a synchronized downregulation of epithelial-integrity and differentiation-related factors, most notably ID2, further supporting the link between SLC25A20-driven metabolic shifts and the observed EMT-like phenotypic remodeling (Fig. 5C, G).

To further determine whether the SLC25A20-regulated transcriptional programs observed in A549 cells were reproducible in an independent LUAD cell model, we examined representative downstream genes in H1299 cells. Consistent with the RNA-seq findings in A549 cells, modulation of SLC25A20 in H1299 cells resulted in similar expression changes in genes associated with lipid metabolism and mitochondrial bioenergetic regulation (Fig. S4A, B). These results indicate that the transcriptional effects associated with SLC25A20 are not restricted to the A549 model, but are reproducible across LUAD cells with different genetic backgrounds. Together with the elevated SLC25A20 expression observed in multiple LUAD cell lines relative to BEAS-2B (Fig. 1D, E), these findings support the broader relevance of SLC25A20-associated metabolic transcriptional regulation in LUAD cells.

### SLC25A20 overexpression is associated with altered lipid metabolism, enhanced mitochondrial respiration, and reduced ROS accumulation in LUAD cells

**Fig. 6:**
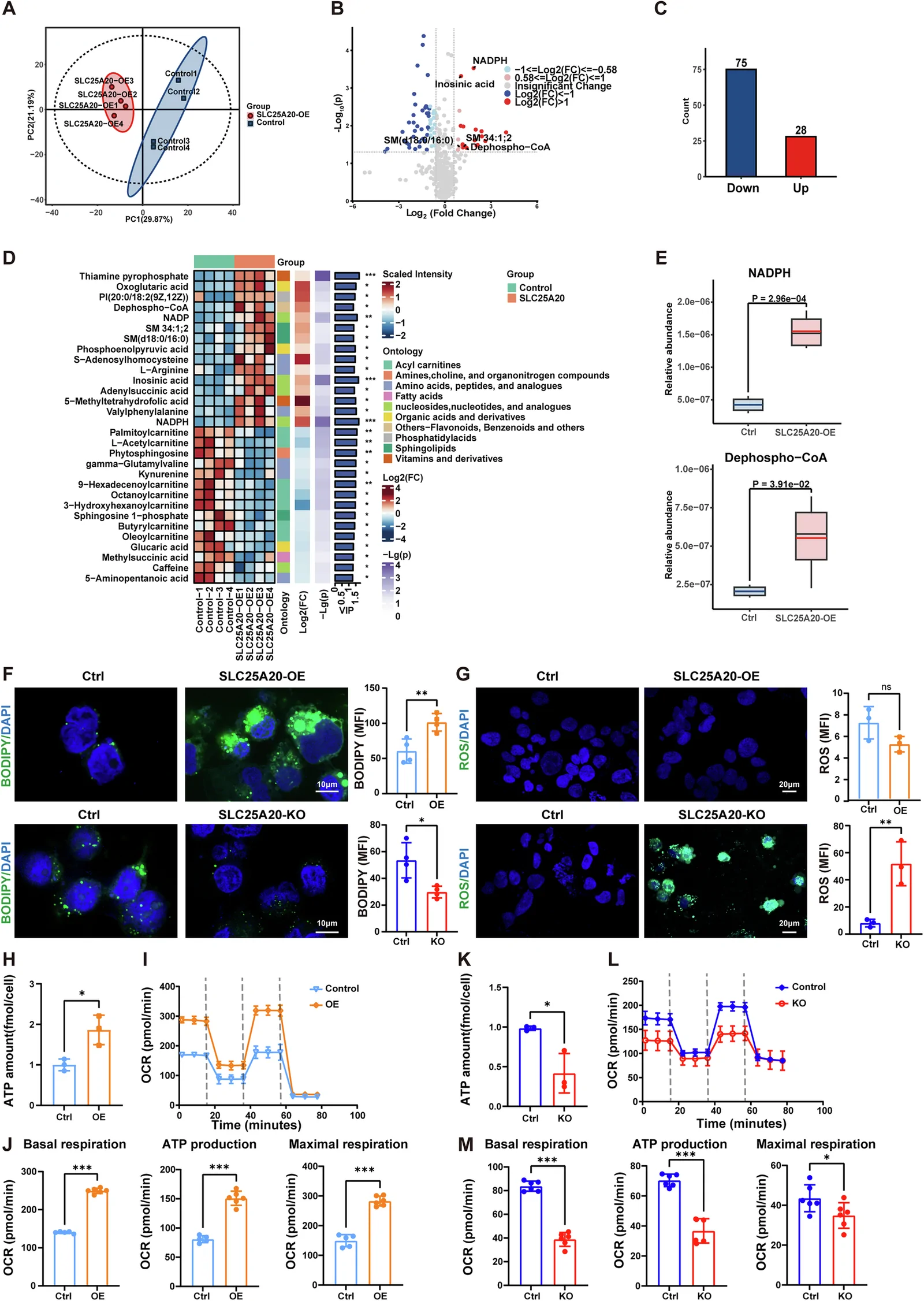
SLC25A20 remodels lipid-associated metabolism and enhances mitochondrial bioenergetics in LUAD cells. **A** PCA score plot of metabolomic profiles from control and SLC25A20-overexpressing A549 cells. Each dot represents an individual sample. **B** Volcano plot showing differential metabolites between control and SLC25A20-overexpressing A549 cells. Red and blue dots indicate significantly increased and decreased metabolites, respectively. Representative differential metabolites are labeled. **C** Number of significantly increased and decreased metabolites identified in SLC25A20-overexpressing A549 cells compared with control cells. **D** Heatmap showing representative differential metabolites between control and SLC25A20-overexpressing A549 cells. Metabolite classes and statistical parameters are annotated on the right. **E** Relative abundance of NADPH and Dephospho-CoA in control and SLC25A20-overexpressing A549 cells. **F** Representative BODIPY 493/503 staining images and quantification of lipid accumulation in SLC25A20-overexpressing and SLC25A20-KO cells compared with their respective control cells. Nuclei were counterstained with DAPI. scale bars: 10 μm. **G** Representative ROS staining images and quantification of intracellular ROS levels in SLC25A20-overexpressing and SLC25A20-KO cells compared with their respective control cells. Nuclei were counterstained with DAPI. scale bars: 20 μm. **H** Intracellular ATP levels in control and SLC25A20-overexpressing A549 cells. **I** Representative oxygen consumption rate profiles of control and SLC25A20-overexpressing A549 cells determined by Seahorse extracellular flux analysis. **J** Quantification of basal respiration, ATP-linked respiration, and maximal respiration from the OCR profiles shown in (**I**). **K** Intracellular ATP levels in control and SLC25A20-KO cells. **L** Representative oxygen consumption rate profiles of control and SLC25A20-KO cells determined by Seahorse extracellular flux analysis. **M** Quantification of basal respiration, ATP-linked respiration, and maximal respiration from the OCR profiles shown in (**L**). Data are presented as mean ± SD from three independent experiments unless otherwise indicated. Statistical significance was determined by two-tailed Student’s *t* test for two-group comparisons and Mann–Whitney *U* test for metabolite abundance comparisons. *P < 0.05, **P < 0.01, ***P < 0.001; ns not significant.

To further investigate the biological processes driven by SLC25A20 and identify potential differential metabolites, we performed quantitative metabolomic analysis (Fig. S5A, B). Principal component analysis (PCA) revealed distinct metabolomic profiles between control and SLC25A20-overexpressing cells (Fig. 6A), and volcano plot analysis identified differential metabolites between the two groups (Fig. 6B). Compared with the control group, the SLC25A20-overexpressing group exhibited 28 upregulated and 75 downregulated metabolites (Fig. 6C). Metabolic pathway enrichment analysis suggested that SLC25A20 overexpression was associated with alterations in steroid/cholesterol biosynthesis, lipid synthesis, and FAO-related metabolic pathways (Fig. 6D; Fig. S5C–L). To further characterize the enriched pathways, we annotated representative differential metabolites according to their corresponding metabolic categories. Cholesterol-related intermediates were assigned to steroid/cholesterol biosynthesis, lipid-associated metabolites were mapped to lipid synthesis or lipid remodeling, and altered acylcarnitine species were interpreted as FAO-related metabolites (Supplementary Table 2). This annotation provides a clearer link between the differential metabolite profile and the pathway-level interpretation of SLC25A20-associated metabolic remodeling.

Given the pivotal role of SLC25A20 in mitochondrial metabolite transport, we further analyzed functional readouts associated with lipid homeostasis and cellular energy status. Notably, Dephospho-CoA, a critical intermediate in CoA biosynthesis and acyl-CoA metabolism, was significantly elevated in SLC25A20-overexpressing cells (Fig. 6E), highlighting a robust expansion of the lipid-related cofactor pool. NADPH abundance was also increased in SLC25A20-overexpressing cells, suggesting a potential enhancement of reducing-equivalent availability (Fig. 6E).

To evaluate the effect of SLC25A20 on lipid metabolism, we performed BODIPY 493/503 staining to detect intracellular neutral lipids and lipid droplet accumulation. Consistent with the transcriptomic profiling results showing upregulation of lipogenic genes including FASN, FADS2, and DHCR7, overexpression of SLC25A20 dramatically boosted neutral lipid accumulation in A549 cells, while SLC25A20 knockout markedly reduced intracellular lipid storage (Fig. 6F). These results indicate that SLC25A20 participates in regulating lipid synthesis and storage in LUAD cells. Combining transcriptomic data and functional experimental results, we propose that SLC25A20 enhances the biosynthetic capacity and metabolic fitness of tumor cells by facilitating the reprogramming of lipid synthesis-related metabolic networks, thereby sustaining the continuous proliferation of LUAD cells. In addition, SLC25A20 overexpression significantly elevated intracellular ATP levels, whereas its knockout impaired ATP production (Fig. 6H, K), further corroborating its vital role in maintaining energy metabolism in tumor cells.

To further evaluate mitochondrial respiratory activity, we performed Seahorse Mito Stress Test analysis. SLC25A20 overexpression increased basal and maximal oxygen consumption rates (Fig. 6I, J), whereas SLC25A20 knockout reduced OCR (Fig. 6L, M), supporting the role of SLC25A20 in enhancing mitochondrial energy metabolism.

Because altered mitochondrial lipid metabolism can also affect cellular redox status, we further measured total cellular ROS levels. ROS levels were reduced in SLC25A20-overexpressing cells but elevated in SLC25A20-KO cells (Fig. 6G). These data indicate that SLC25A20 expression is associated with changes in cellular redox status. Together with the ATP and OCR measurements, these findings suggest that SLC25A20 affects both mitochondrial bioenergetic output and redox homeostasis; Nevertheless, changes in intracellular ROS constitute a distinct metabolic feature and are not directly attributable to variations in ATP synthesis.

Collectively, these findings suggest that SLC25A20 is associated with lipid metabolism remodeling, enhanced mitochondrial respiration, increased ATP production, and reduced ROS accumulation in LUAD cells. These metabolic changes may contribute to LUAD cell proliferation and survival by supporting cellular bioenergetics while helping maintain redox homeostasis.

### SLC25A20 promotes LUAD cell proliferation and survival partly through FAO-related mitochondrial bioenergetics

To determine whether FAO-related mitochondrial bioenergetics contributes to SLC25A20-driven malignant phenotypes, we treated SLC25A20-overexpressing A549 cells with Etomoxir, a pharmacological inhibitor of CPT1-dependent fatty acid entry into mitochondria. This strategy allowed us to assess whether the metabolic and functional advantages conferred by SLC25A20 overexpression were sensitive to pharmacological inhibition of FAO. Because parental A549 cells retain endogenous SLC25A20 expression, Etomoxir-treated A549 cells were also analyzed as an additional reference to evaluate the basal dependence of LUAD cells on FAO-related metabolism.

**Fig. 7:**
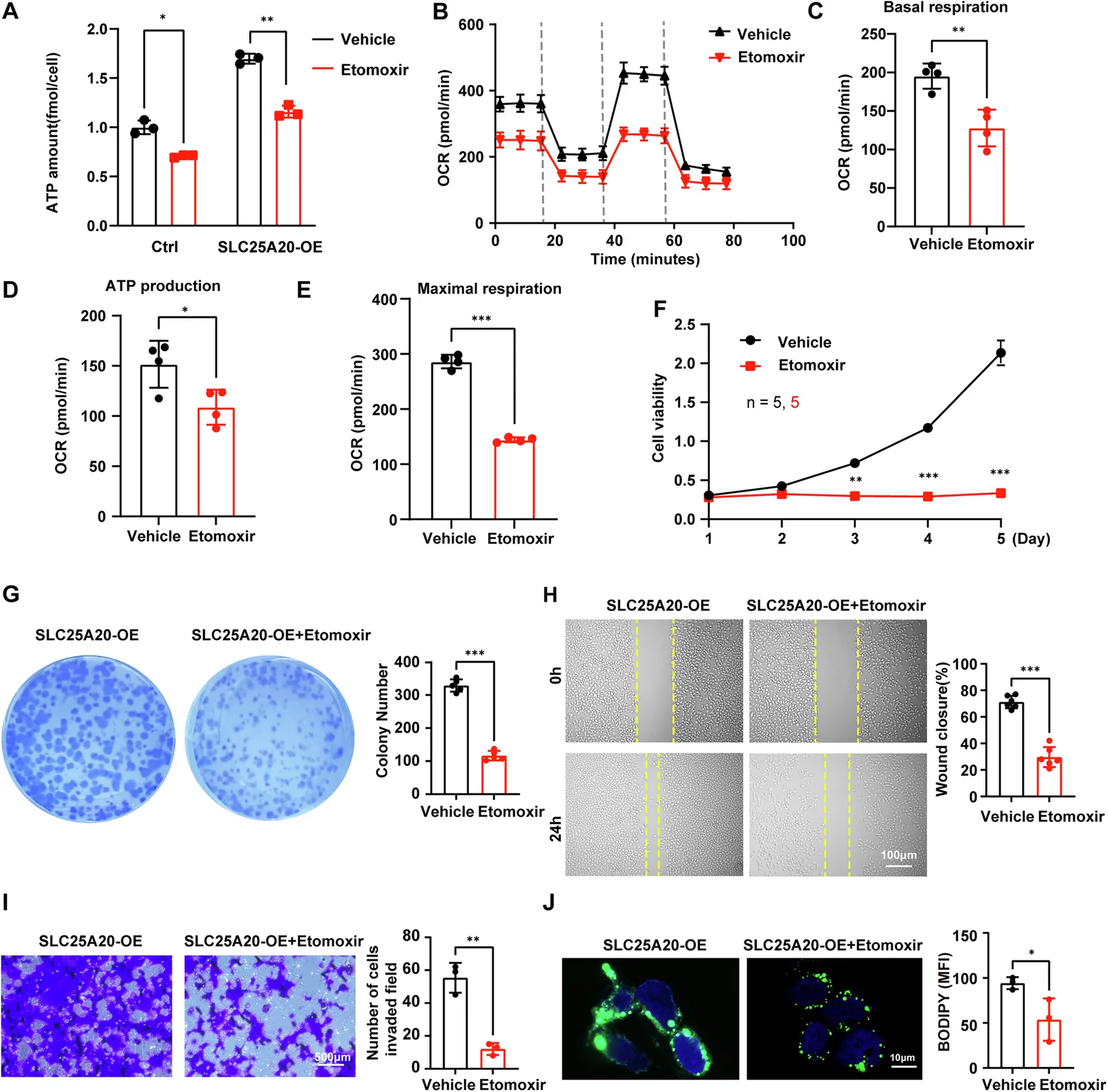
Etomoxir suppresses SLC25A20-driven bioenergetic, proliferative, and invasive phenotypes in LUAD cells. **A** Intracellular ATP levels in SLC25A20-overexpressing A549 cells treated with vehicle or Etomoxir. **B** Oxygen consumption rate (OCR) profiles of SLC25A20-overexpressing A549 cells treated with vehicle or Etomoxir, as determined by Seahorse extracellular flux analysis. **C–E** Quantification of basal respiration, ATP-linked respiration, and maximal respiration from the OCR profiles shown in (**B**). **F** Cell viability of SLC25A20-overexpressing A549 cells treated with vehicle or Etomoxir at the indicated time points. **G** Colony formation assays of SLC25A20-overexpressing A549 cells treated with vehicle or Etomoxir. Representative images and quantification of colony numbers are shown. **H** Wound-healing assays of SLC25A20-overexpressing A549 cells treated with vehicle or Etomoxir. Representative images at 0 and 24 h and quantification of wound closure are shown. Scale bar: 100 μm. **I** Matrigel-coated Transwell invasion assays of SLC25A20-overexpressing A549 cells treated with vehicle or Etomoxir. Representative images and quantification of invaded cells per field are shown. Scale bar: 500 μm. **J** Representative BODIPY 493/503 staining images and quantification of neutral lipid accumulation in SLC25A20-overexpressing A549 cells treated with vehicle or Etomoxir. Nuclei were counterstained with DAPI. Scale bar: 10 μm. Data are presented as mean ± SD from three independent experiments. Statistical significance was determined by two-tailed Student’s *t* test for two-group comparisons and two-way ANOVA for cell viability curves. *P < 0.05, **P < 0.01, ***P < 0.001.

Intracellular ATP levels were first measured using a bioluminescence-based ATP assay. SLC25A20 overexpression increased intracellular ATP abundance, whereas Etomoxir markedly reduced ATP levels in SLC25A20-overexpressing A549 cells (Fig. 7A). These findings suggest that the enhanced bioenergetic activity driven by SLC25A20 is at least partly dependent on FAO-related metabolism. Consistently, Etomoxir also suppressed ATP-associated metabolic activity and cell growth in parental A549 cells, although the inhibitory effect was more pronounced in the SLC25A20-overexpressing context (Fig. 7A).

We next examined mitochondrial respiratory activity using Seahorse extracellular flux analysis. Etomoxir treatment significantly decreased the oxygen consumption rate (OCR) in SLC25A20-overexpressing A549 cells and reduced basal respiration, ATP-linked respiration, and maximal respiration (Fig. 7B–E). These results indicate that inhibition of CPT1-dependent FAO compromises the mitochondrial respiratory advantage conferred by SLC25A20 overexpression. In parallel, Etomoxir reduced OCR and respiratory parameters in parental A549 cells, further supporting the contribution of FAO-related mitochondrial respiration to LUAD cell bioenergetics under basal conditions (Fig. S6C).

Functionally, Etomoxir attenuated SLC25A20-driven malignant phenotypes in A549 cells. In SLC25A20-overexpressing cells, Etomoxir reduced cell viability, colony formation, wound closure, and Matrigel invasion (Fig. 7F–I). BODIPY 493/503 staining further showed that Etomoxir decreased neutral lipid accumulation in SLC25A20-overexpressing cells, supporting the involvement of FAO-related lipid metabolic remodeling in this phenotype (Fig. 7J). Similar inhibitory effects on proliferation, clonogenic growth, migration, invasion, and neutral lipid accumulation were also observed in parental A549 cells treated with Etomoxir (Fig. S6A, E, G, I, K). Notably, Etomoxir attenuated but did not completely abolish SLC25A20-associated phenotypes, suggesting that SLC25A20 may promote LUAD progression through FAO-dependent as well as additional metabolic mechanisms.

To further evaluate whether FAO inhibition is effective in an endogenous SLC25A20-high setting, we treated H1975 cells with Etomoxir. H1975 cells showed relatively high endogenous SLC25A20 expression in our cell-line screening. Similar to the findings in SLC25A20-overexpressing A549 cells, Etomoxir reduced OCR, cell proliferation, colony formation, migration, invasion, and neutral lipid accumulation in H1975 cells (Fig. S6B, D, F, H, J, L). These results indicate that the effect of FAO inhibition is not restricted to an overexpression model but is also observed in LUAD cells with relatively high endogenous SLC25A20 expression.

**Fig. 8:**
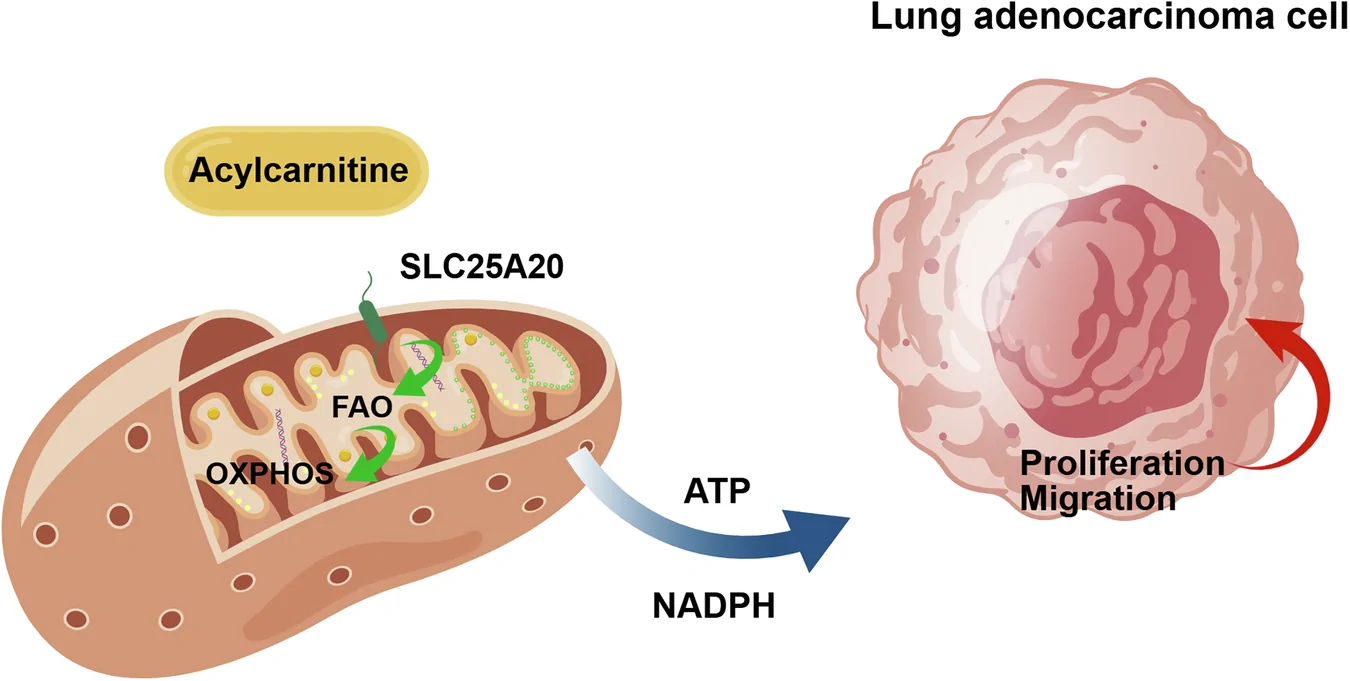
Proposed model for the role of SLC25A20 in LUAD metabolic reprogramming and tumor progression. SLC25A20 facilitates mitochondrial acylcarnitine transport and enhances fatty acid oxidation (FAO), thereby supporting oxidative phosphorylation (OXPHOS), ATP production, and NADPH-associated redox homeostasis. This metabolic remodeling promotes LUAD cell proliferation and migration while reducing ROS accumulation and apoptosis, ultimately contributing to tumor progression. Created with BioGDP.com [42].

Together, these findings support that SLC25A20 promotes LUAD cell proliferation, survival, migration, and invasion at least in part by enhancing FAO-related mitochondrial bioenergetics and ATP production. Given that Etomoxir targets CPT1-dependent fatty acid entry into mitochondria, whereas SLC25A20 mediates acylcarnitine/carnitine exchange across the inner mitochondrial membrane, these data should be interpreted as evidence for the involvement of FAO-related metabolism rather than proof that all SLC25A20-driven effects are exclusively FAO-dependent. Combined with the observed reduction in ROS accumulation upon SLC25A20 overexpression, these results suggest that SLC25A20 supports LUAD progression by coordinating mitochondrial energy production, lipid metabolic remodeling, and redox homeostasis (Fig. 8).

## Discussion

Our study identifies SLC25A20 as a key metabolic driver that promotes tumor progression in lung adenocarcinoma (LUAD) by reprogramming fatty acid metabolism. We demonstrate that SLC25A20 is upregulated in LUAD tissues and cell lines, and that its elevated expression is associated with adverse clinical outcomes. Functional gain- and loss-of-function experiments further revealed that SLC25A20 enhances tumor cell proliferation, colony formation, migration, invasion, and in vivo tumor growth. Mechanistically, SLC25A20 promotes fatty acid oxidation (FAO)-related mitochondrial metabolism, supports ATP production, reduces intracellular reactive oxygen species (ROS) accumulation, and suppresses apoptosis. It is noteworthy that the prognostic value of SLC25A20 appears to be context-dependent. Although SLC25A20 expression was not significantly associated with overall survival in the unstratified TCGA-LUAD cohort, subtype-specific analyses using the GEPIA2 platform revealed significant associations with survival outcomes in the TRU and PP molecular subtypes. These findings suggest that the clinical significance of SLC25A20 may be influenced by the molecular and metabolic heterogeneity of LUAD, and support its role as a context-specific prognostic indicator rather than a universal prognostic biomarker. Collectively, our data indicate that SLC25A20 enables LUAD cells to acquire a metabolic advantage that supports both tumor growth and malignant progression. Together with the observed elevation of SLC25A20 in paired clinical LUAD samples, these observations suggest that SLC25A20 may help identify a metabolically relevant subgroup of LUAD tumors, although this possibility requires further validation in larger clinical cohorts.

One of the key findings of this study is that SLC25A20 enhances FAO-associated mitochondrial bioenergetics in LUAD cells. As a mitochondrial carnitine-acylcarnitine translocase, SLC25A20 is positioned to facilitate the mitochondrial transport of long-chain acylcarnitines, thereby supporting fatty acid β-oxidation. Consistently, SLC25A20 overexpression increased intracellular ATP levels and ATP-linked mitochondrial respiration, whereas SLC25A20 depletion reduced ATP levels and oxygen consumption rate (OCR). These results indicate that SLC25A20 contributes to the bioenergetic adaptation of LUAD cells by enhancing mitochondrial fatty acid utilization. Importantly, validation in H1299 cells showed that representative SLC25A20-associated transcriptional changes were also observed in an independent LUAD cell background. This cross-model consistency supports the view that SLC25A20-associated lipid metabolic and mitochondrial bioenergetic remodeling is not limited to a single A549 overexpression model. Nevertheless, because these analyses were performed in selected LUAD cell models, further studies across larger cell-line panels and patient-derived models will be useful to define the extent to which these transcriptional programs correlate with endogenous SLC25A20 abundance. Notably, the increased intracellular lipid content observed in SLC25A20-overexpressing cells, together with the upregulation of lipogenic genes such as FASN, SCD, and FADS2, suggests that SLC25A20 does not merely promote lipid catabolism but may coordinate a high-turnover lipid metabolic state. In this model, both lipid synthesis and lipid utilization may be enhanced to meet the membrane, signaling, and energy demands of rapidly proliferating tumor cells [34–36].

In addition to enhancing bioenergetic capacity, SLC25A20 also appears to reshape the redox state of LUAD cells. SLC25A20 overexpression reduced total cellular ROS accumulation, whereas SLC25A20 knockout increased ROS levels and promoted apoptosis. We do not interpret these ROS changes as a direct or linear consequence of ATP production. Rather, ATP production and ROS accumulation represent two related but distinct functional outputs of SLC25A20-associated metabolic remodeling. Metabolomic profiling showed increased relative NADPH abundance in SLC25A20-overexpressing cells, which may reflect enhanced reducing-equivalent availability and could contribute to antioxidant buffering. However, further direct biochemical assays will be required to define the precise redox mechanism [34, 37, 38].

Intriguingly, our transcriptomic analysis revealed significant downregulation of HMOX1, a canonical stress-responsive antioxidant enzyme, in SLC25A20-overexpressing cells. Because HMOX1 is typically induced in response to oxidative stress, its decrease in our model may reflect a reduced requirement for stress-responsive antioxidant programs in SLC25A20-high cells. One possible explanation is that SLC25A20-associated metabolic remodeling improves redox balance by enhancing FAO-linked reducing capacity and limiting oxidative stress. However, whether SLC25A20 directly affects mitochondrial ROS generation, electron leakage, or electron transport chain efficiency requires further investigation. Together, these findings suggest that SLC25A20 helps LUAD cells maintain a “high-energy, low-stress” metabolic state that favors survival and tumor progression.

The contribution of FAO-related metabolism to SLC25A20-driven phenotypes was further supported by Etomoxir treatment, a pharmacological inhibitor of CPT1-dependent fatty acid entry into mitochondria. In SLC25A20-overexpressing A549 cells, Etomoxir reduced intracellular ATP levels, decreased mitochondrial respiration, and attenuated the proliferative, migratory, and invasive advantages associated with SLC25A20 overexpression. Similar inhibitory effects were also observed in H1975 cells, which express relatively high endogenous SLC25A20, supporting the relevance of FAO-related metabolism in SLC25A20-high LUAD contexts. However, Etomoxir treatment and SLC25A20 loss-of-function should not be considered identical interventions. Etomoxir inhibits CPT1-dependent fatty acid entry into mitochondria at the outer mitochondrial membrane, whereas SLC25A20 mediates acylcarnitine/carnitine exchange across the inner mitochondrial membrane. Therefore, the Etomoxir experiments support the involvement of FAO-related metabolism in SLC25A20-associated metabolic and functional phenotypes, but they do not prove that all SLC25A20-driven effects are entirely FAO-dependent. The partial attenuation of SLC25A20-associated phenotypes by Etomoxir further suggests that additional metabolic mechanisms may also contribute to SLC25A20-driven LUAD progression [37].

The link between SLC25A20-mediated metabolic remodeling and enhanced malignancy may involve several non-mutually exclusive mechanisms. First, cell migration and invasion are energy-demanding processes that require cytoskeletal remodeling, membrane protrusion formation, focal adhesion turnover, and extracellular matrix degradation. Enhanced FAO-associated mitochondrial respiration may provide ATP to support these processes, consistent with previous evidence that ATP utilization is dynamically regulated during metastatic cell migration [39]. Second, reduced ROS accumulation in SLC25A20-overexpressing cells may help maintain cellular fitness during invasive growth, as cancer cells frequently adapt to oxidative stress by increasing NADPH-dependent antioxidant capacity [38]. Third, the synchronized upregulation of lipid biosynthesis genes, including FASN, SCD, and FADS2, suggests a coupling between FAO and lipid anabolism, which may sustain membrane remodeling and signaling lipid pools necessary for EMT-associated migratory [35, 36]. Further studies will be required to identify the direct downstream effectors linking SLC25A20-mediated metabolism to cytoskeletal remodeling, focal adhesion dynamics, and extracellular matrix invasion.

Our work adds to the growing body of evidence that mitochondrial transporters are active regulators of cancer metabolism rather than passive carriers. Similar to other SLC25 family members involved in citrate export, malate transport, mitochondrial redox balance, and metabolic adaptation, SLC25A20 may represent a critical nexus connecting nutrient utilization with oncogenic phenotypes [30, 40, 41]. Given the growing interest in targeting mitochondrial metabolism in cancer, SLC25A20 may serve as a potential metabolic vulnerability in LUAD, particularly in tumors with high dependence on FAO-associated bioenergetics.

Several limitations should be acknowledged. First, although our findings were validated in multiple LUAD cell models and paired clinical samples, additional patient-derived xenograft models, organoid systems, and larger clinically annotated cohorts are needed to confirm the clinical relevance of SLC25A20. In addition, the prognostic associations observed in transcriptomic datasets may not fully reflect the magnitude of changes observed at the protein level. The more pronounced differences in SLC25A20 protein expression detected in our clinical samples suggest that discrepancies between mRNA and protein abundance may partially contribute to the variations observed across different clinical datasets. Moreover, given the subtype-dependent prognostic associations observed in public datasets, larger molecularly stratified clinical cohorts will be required to further define the prognostic relevance of SLC25A20 across distinct LUAD subtypes. In particular, larger independent cohorts will be required to define the frequency, clinicopathological features, and therapeutic relevance of SLC25A20-high LUAD more precisely. Second, although our data suggest a link between SLC25A20, FAO, and redox homeostasis, direct measurements of NADPH levels, GSH/GSSG balance, mitochondrial ROS, and mitochondrial respiratory chain efficiency would further clarify the underlying antioxidant mechanisms. Third, because Etomoxir and SLC25A20 knockout affect FAO-related metabolism at different steps, additional genetic and pharmacological approaches will be required to define the precise dependence of SLC25A20-driven phenotypes on FAO. Finally, the potential roles of SLC25A20 in therapy resistance, immune evasion, and tumor microenvironment remodeling remain to be investigated.

In conclusion, our study identifies SLC25A20 as a previously underappreciated metabolic driver of LUAD progression. By enhancing FAO-associated bioenergetics, maintaining redox balance, and supporting malignant phenotypes, SLC25A20 enables LUAD cells to acquire a significant metabolic advantage. Targeting SLC25A20 or its associated metabolic program may provide a promising therapeutic strategy for patients with SLC25A20-high lung adenocarcinoma.

## Supplementary information

Supplementary Material

Supplementary material

aj-checklist

## Data Availability

The RNA sequencing data generated in this study have been deposited in the NCBI Sequence Read Archive (SRA) under BioProject accession number PRJNA1461143. The corresponding BioSample records (SAMN57691913–SAMN57691918) are available through the NCBI BioSample database. All data supporting the findings of this study are publicly available at the time of publication.
