# Supplementary material for: SLC25A20 promotes tumor growth by reprogramming energy metabolism in lung adenocarcinoma
Source: Cell Death Dis. 2026 Jul 23;17(1):893. doi: 10.1038/s41419-026-09119-9 (PMC13680618; doi:10.1038/s41419-026-09119-9)
Supplement: Supplementary file 1 [file 41419_2026_9119_MOESM2_ESM.pdf]

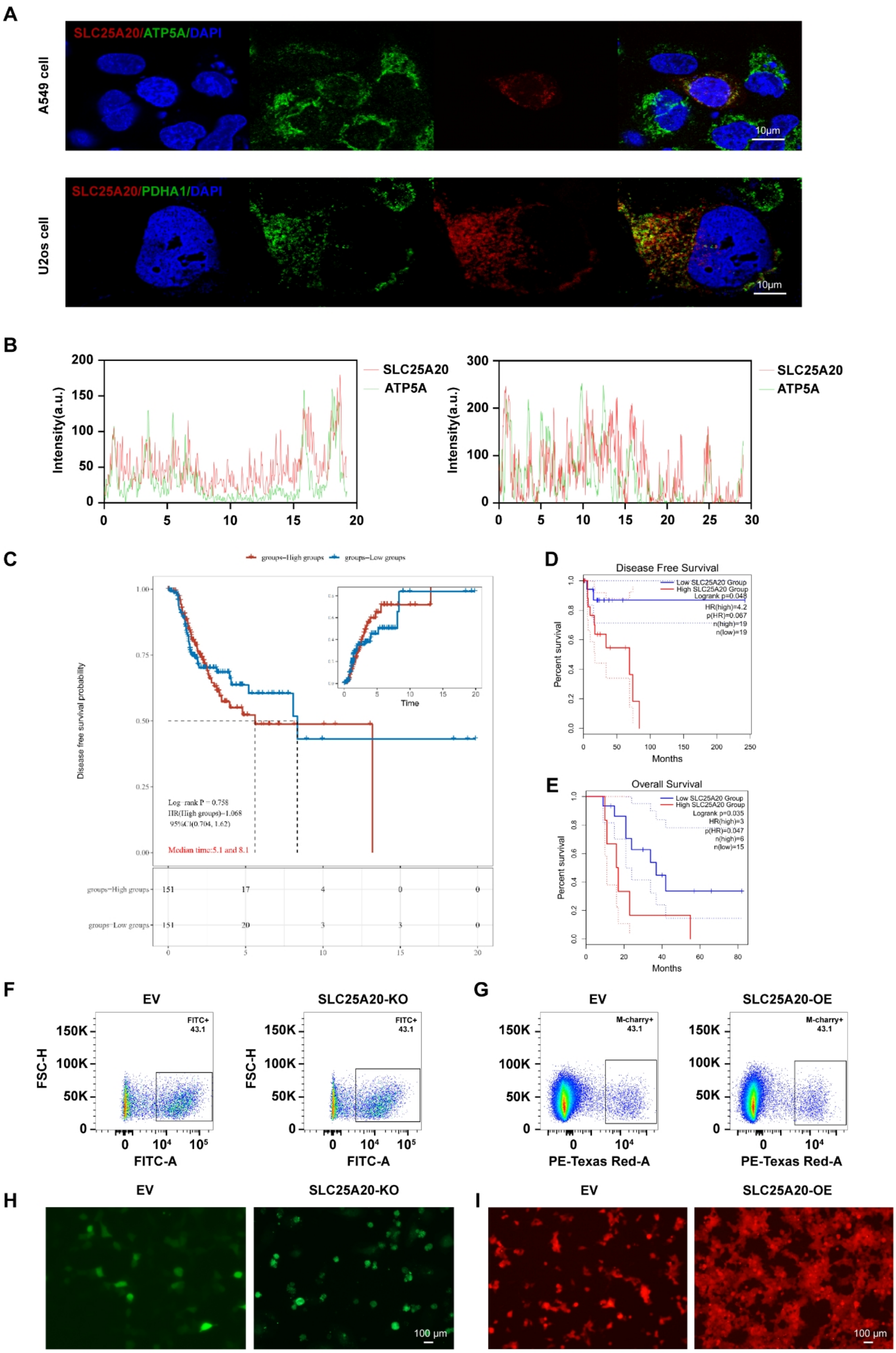

A
A549 cell
SLC25A20/ATP5A/DAPI
10μm
U2os cell
SLC25A20/PDHA1/DAPI
10μm
B
SLC25A20
ATP5A
Intensity(a.u.)
C
groups=High groups
groups=Low groups
Disease free survival probability
Time
Log-rank P = 0.758
HR(High groups)=1.068
95%CI(0.704, 1.62)
Median time:5.1 and 8.1
D
Disease Free Survival
Low SLC25A20 Group
High SLC25A20 Group
Logrank p=0.048
HR(high)=4.2
p(HR)=0.067
n(high)=19
n(low)=19
Percent survival
Months
E
Overall Survival
Low SLC25A20 Group
High SLC25A20 Group
Logrank p=0.035
HR(high)=3
p(HR)=0.047
n(high)=6
n(low)=15
Percent survival
Months
F
EV
SLC25A20-KO
FITC+
43.1
FSC-H
FITC-A
G
EV
SLC25A20-OE
M-charry+
43.1
PE-Texas Red-A
H
EV
SLC25A20-KO
100 μm
I
EV
SLC25A20-OE
100 μm

Supplementary Figure 1. Establishment and validation of SLC25A20-knockout and SLC25A20-overexpressing cell models.

A. Representative immunofluorescence images showing the subcellular localization of SLC25A20 in A549 and U2OS cells. SLC25A20 was co-stained with the mitochondrial markers ATP5A or PDHA1, and nuclei were counterstained with DAPI. Scale bars, 10 μm. **B.** Line-scan fluorescence intensity profiles showing the spatial overlap between SLC25A20 and the mitochondrial marker ATP5A in the representative regions shown in **A**. **C.** Kaplan – Meier analysis of disease-free survival (DFS) in the TCGA-LUAD cohort stratified by median SLC25A20 expression. **D.** Kaplan–Meier analysis of disease-free survival (DFS) according to SLC25A20 expression in the terminal respiratory unit (TRU) subtype of LUAD from the GEPIA2 database. **E.** Kaplan–Meier analysis of overall survival (OS) according to SLC25A20 expression in the proximal proliferation (PP) subtype of LUAD from the GEPIA2 database. **F, G.** Flow cytometry analysis of reporter fluorescence in EV and SLC25A20-KO/OE cells. **H.** Representative fluorescence microscopy images confirming SLC25A20 knockout in EV and SLC25A20-KO H1975 cell line. Scale bar, 100 μm. **I.** Representative fluorescence microscopy images confirming SLC25A20 overexpression in EV and SLC25A20-OE A549 cell line. Scale bar, 100 μm.

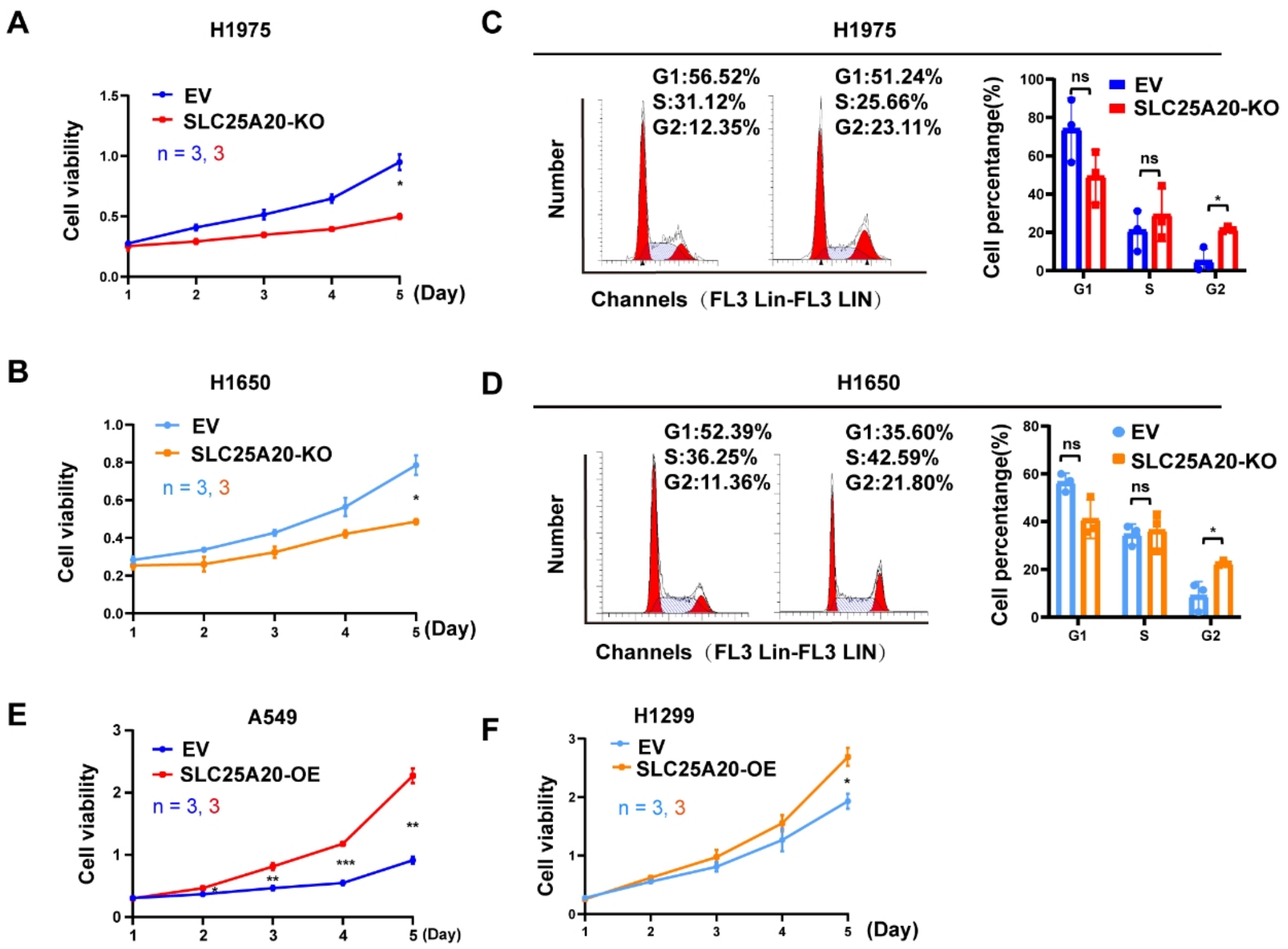
A
H1975
EV
SLC25A20-KO
n = 3, 3
Cell viability
(Day)
B
H1650
EV
SLC25A20-KO
n = 3, 3
Cell viability
(Day)
C
H1975
G1:56.52%
S:31.12%
G2:12.35%
G1:51.24%
S:25.66%
G2:23.11%
Number
Channels（FL3 Lin-FL3 LIN）
Cell percentange(%)
EV
SLC25A20-KO
ns
G1
S
G2
D
H1650
G1:52.39%
S:36.25%
G2:11.36%
G1:35.60%
S:42.59%
G2:21.80%
Number
Channels（FL3 Lin-FL3 LIN）
Cell percentange(%)
EV
SLC25A20-KO
E
A549
EV
SLC25A20-OE
n = 3, 3
Cell viability
(Day)
F
H1299
EV
SLC25A20-OE
n = 3, 3
Cell viability
(Day)

Supplementary Figure 2. SLC25A20 regulates LUAD cell viability and cell-cycle progression. **A.** Cell viability of EV and SLC25A20-KO H1975 cells measured at the indicated time points. **B.** Representative flow cytometry histograms and quantification of cell-cycle distribution in EV and SLC25A20-KO H1975 cells following PI staining. **C.** Cell viability of EV and SLC25A20-KO H1650 cells measured at the indicated time points. **D.** Representative flow cytometry histograms and quantification of cell-cycle distribution in EV and SLC25A20-KO H1650 cells following PI staining. **E.** Cell viability of EV and SLC25A20-overexpressing A549 cells measured at the indicated time points. **F.** Cell viability of EV and SLC25A20-overexpressing H1299 cells measured at the indicated time points. Data are presented as mean ± SD from three independent experiments. Statistical significance was determined by two-way ANOVA for cell viability curves and two-tailed Student's t-test for two-group comparisons in cell-cycle analyses. *$P < 0.05$; ns, not significant.

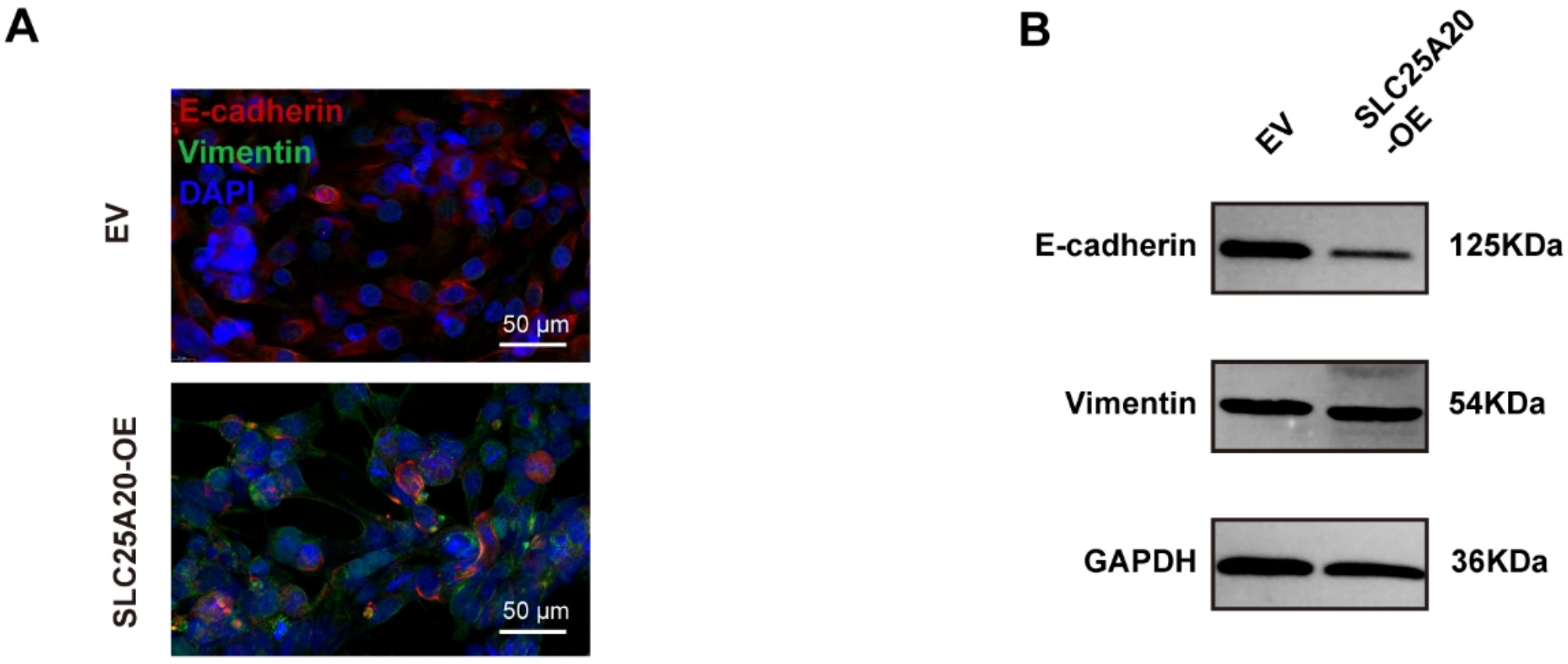
A
E-cadherin
Vimentin
DAPI
EV
SLC25A20-OE
50 μm
50 μm
B
EV
SLC25A20-OE
E-cadherin
125KDa
Vimentin
54KDa
GAPDH
36KDa

Supplementary Figure 3. SLC25A20 overexpression promotes EMT marker remodeling in A549 cells.

A. Representative immunofluorescence images showing E-cadherin and Vimentin expression in EV and SLC25A20-overexpressing LUAD cells. E-cadherin or Vimentin is shown in red, and nuclei were counterstained with DAPI. Scale bars, 50 μm. **B.** Western blot analysis of E-cadherin and Vimentin protein expression in EV and SLC25A20-overexpressing LUAD cells. GAPDH was used as the loading control.

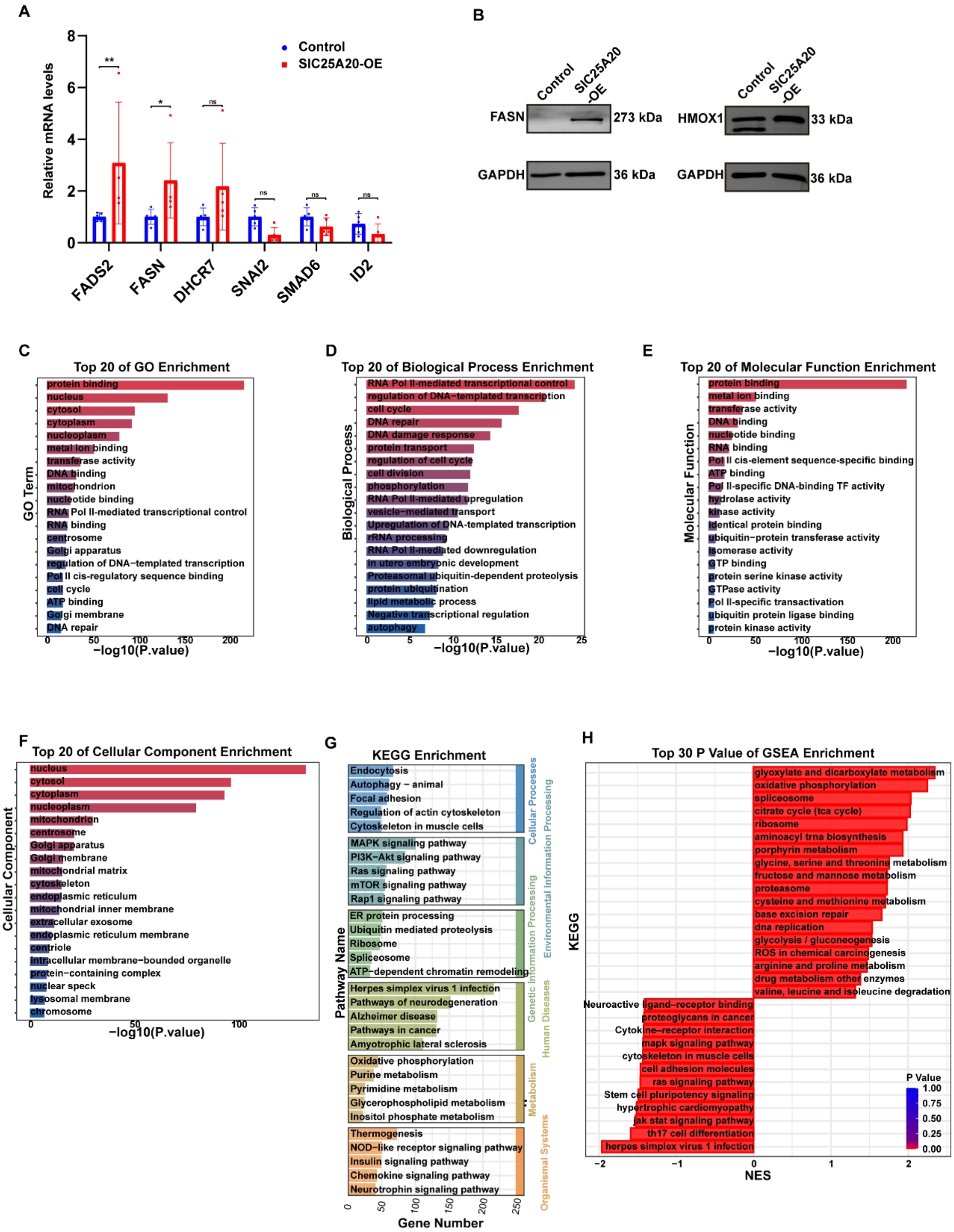
A
Relative mRNA levels
Control
SIC25A20-OE
**
*
ns
FADS2
FASN
DHCR7
SNAI2
SMAD6
ID2
B
Control
SIC25A20-OE
FASN
273 kDa
GAPDH
36 kDa
HMOX1
33 kDa
GAPDH
36 kDa
C
Top 20 of GO Enrichment
GO Term
protein binding
nucleus
cytosol
cytoplasm
nucleoplasm
metal ion binding
transferase activity
DNA binding
mitochondrion
nucleotide binding
RNA Pol II-mediated transcriptional control
RNA binding
centrosome
Golgi apparatus
regulation of DNA−templated transcription
Pol II cis-regulatory sequence binding
cell cycle
ATP binding
Golgi membrane
DNA repair
−log10(P.value)
D
Top 20 of Biological Process Enrichment
Biological Process
RNA Pol II-mediated transcriptional control
regulation of DNA−templated transcription
cell cycle
DNA repair
DNA damage response
protein transport
regulation of cell cycle
cell division
phosphorylation
RNA Pol II-mediated upregulation
vesicle−mediated transport
Upregulation of DNA-templated transcription
rRNA processing
RNA Pol II-mediated downregulation
in utero embryonic development
Proteasomal ubiquitin-dependent proteolysis
protein ubiquitination
lipid metabolic process
Negative transcriptional regulation
autophagy
−log10(P.value)
E
Top 20 of Molecular Function Enrichment
Molecular Function
protein binding
metal ion binding
transferase activity
DNA binding
nucleotide binding
RNA binding
Pol II cis-element sequence-specific binding
ATP binding
Pol II-specific DNA-binding TF activity
hydrolase activity
kinase activity
identical protein binding
ubiquitin−protein transferase activity
isomerase activity
GTP binding
protein serine kinase activity
GTPase activity
Pol II-specific transactivation
ubiquitin protein ligase binding
protein kinase activity
−log10(P.value)
F
Top 20 of Cellular Component Enrichment
Cellular Component
nucleus
cytosol
cytoplasm
nucleoplasm
mitochondrion
centrosome
Golgi apparatus
Golgi membrane
mitochondrial matrix
cytoskeleton
endoplasmic reticulum
mitochondrial inner membrane
extracellular exosome
endoplasmic reticulum membrane
centriole
intracellular membrane−bounded organelle
protein−containing complex
nuclear speck
lysosomal membrane
chromosome
−log10(P.value)
G
KEGG Enrichment
Pathway Name
Endocytosis
Autophagy − animal
Focal adhesion
Regulation of actin cytoskeleton
Cytoskeleton in muscle cells
MAPK signaling pathway
PI3K−Akt signaling pathway
Ras signaling pathway
mTOR signaling pathway
Rap1 signaling pathway
ER protein processing
Ubiquitin mediated proteolysis
Ribosome
Spliceosome
ATP−dependent chromatin remodeling
Herpes simplex virus 1 infection
Pathways of neurodegeneration
Alzheimer disease
Pathways in cancer
Amyotrophic lateral sclerosis
Oxidative phosphorylation
Purine metabolism
Pyrimidine metabolism
Glycerophospholipid metabolism
Inositol phosphate metabolism
Thermogenesis
NOD−like receptor signaling pathway
Insulin signaling pathway
Chemokine signaling pathway
Neurotrophin signaling pathway
Cellular Processes
Environmental Information Processing
Genetic Information Processing
Human Diseases
Metabolism
Organismal Systems
Gene Number
H
Top 30 P Value of GSEA Enrichment
KEGG
glyoxylate and dicarboxylate metabolism
oxidative phosphorylation
spliceosome
citrate cycle (tca cycle)
ribosome
aminoacyl trna biosynthesis
porphyrin metabolism
glycine, serine and threonine metabolism
fructose and mannose metabolism
proteasome
cysteine and methionine metabolism
base excision repair
dna replication
glycolysis / gluconeogenesis
ROS in chemical carcinogenesis
arginine and proline metabolism
drug metabolism other enzymes
valine, leucine and isoleucine degradation
Neuroactive ligand−receptor binding
proteoglycans in cancer
Cytokine−receptor interaction
mapk signaling pathway
cytoskeleton in muscle cells
cell adhesion molecules
ras signaling pathway
Stem cell pluripotency signaling
hypertrophic cardiomyopathy
jak stat signaling pathway
th17 cell differentiation
herpes simplex virus 1 infection
P Value
NES

Supplementary Figure 4. Validation and functional enrichment analysis of SLC25A20-associated differentially expressed genes.

A. qRT-PCR validation of selected differentially expressed genes in control and SLC25A20-overexpressing cells. **B.** Western blot validation of FASN and HMOX1 protein expression in control and SLC25A20-overexpressing cells. GAPDH was used as the loading control. **C.** Top 20 enriched Gene Ontology terms among SLC25A20-associated differentially expressed genes, ranked by −log10(P value). **D.** Top 20 enriched Gene Ontology Biological Process terms among SLC25A20-associated differentially expressed genes. **E.** Top 20 enriched Gene Ontology Molecular Function terms among SLC25A20-associated differentially expressed genes. **F.** Top 20 enriched Gene Ontology Cellular Component terms among SLC25A20-associated differentially expressed genes. **G.** KEGG pathway enrichment analysis of SLC25A20-associated differentially expressed genes. Pathways are grouped according to KEGG functional categories, and the x-axis indicates the number of genes enriched in each pathway. **H.** Top 30 enriched pathways identified by GSEA. Normalized enrichment scores (NES) indicate the direction and magnitude of pathway enrichment. Data are presented as mean ± SD from three independent experiments where applicable. Statistical significance was determined by two-tailed Student's t-test for two-group comparisons. *$P < 0.05$.

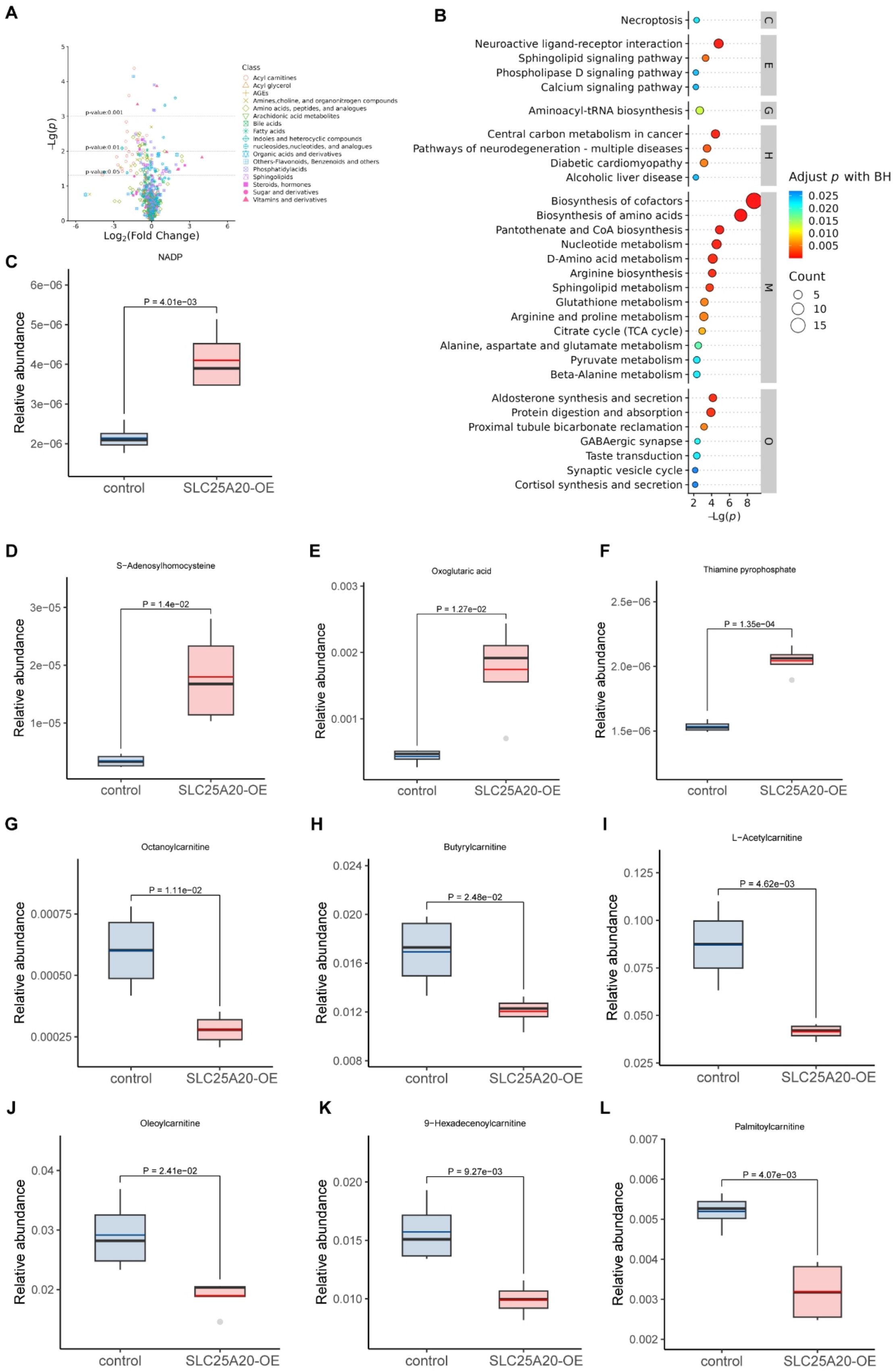

A
Class
-Lg(p)
Log2(Fold Change)
p-value:0.001
p-value:0.01
p-value:0.05
B
Necroptosis
Neuroactive ligand-receptor interaction
Sphingolipid signaling pathway
Phospholipase D signaling pathway
Calcium signaling pathway
Aminoacyl-tRNA biosynthesis
Central carbon metabolism in cancer
Pathways of neurodegeneration - multiple diseases
Diabetic cardiomyopathy
Alcoholic liver disease
Biosynthesis of cofactors
Biosynthesis of amino acids
Pantothenate and CoA biosynthesis
Nucleotide metabolism
D-Amino acid metabolism
Arginine biosynthesis
Sphingolipid metabolism
Glutathione metabolism
Arginine and proline metabolism
Citrate cycle (TCA cycle)
Alanine, aspartate and glutamate metabolism
Pyruvate metabolism
Beta-Alanine metabolism
Aldosterone synthesis and secretion
Protein digestion and absorption
Proximal tubule bicarbonate reclamation
GABAergic synapse
Taste transduction
Synaptic vesicle cycle
Cortisol synthesis and secretion
C
E
G
H
M
O
-Lg(p)
Adjust p with BH
0.025
0.020
0.015
0.010
0.005
Count
5
10
15
C
NADP
P = 4.01e-03
Relative abundance
control
SLC25A20-OE
D
S-Adenosylhomocysteine
P = 1.4e-02
E
Oxoglutaric acid
P = 1.27e-02
F
Thiamine pyrophosphate
P = 1.35e-04
G
Octanoylcarnitine
P = 1.11e-02
H
Butyrylcarnitine
P = 2.48e-02
I
L-Acetylcarnitine
P = 4.62e-03
J
Oleoylcarnitine
P = 2.41e-02
K
9-Hexadecenoylcarnitine
P = 9.27e-03
L
Palmitoylcarnitine
P = 4.07e-03

Supplementary Figure.5

Metabolomic analysis reveals altered metabolic profiles in SLC25A20-overexpressing cells

A. Volcano plot of differentially altered metabolites. This plot visualizes the fold change (x-axis, log2 scale) versus statistical significance (y-axis, -log10 P-value) of all detected metabolites between *SLC25A20*-overexpressing cells and control cells. Metabolites are color-coded by their super-class classification (e.g., lipids, amino acids, carbohydrates), with significantly altered metabolites (e.g., $|\log2\ \text{fold change}| > 0.5$ and $P < 0.05$) being prominent. **B.** Metabolite set enrichment analysis (MSEA) of altered metabolic pathways. This bubble plot displays the significantly enriched metabolic pathways identified by MSEA. The x-axis represents the -log10(P-value), indicating the statistical significance of enrichment. The size of the bubbles corresponds to the number of metabolites found in each pathway (Count), and the color indicates the adjusted P-value (P-adjust with BH), with warmer colors representing higher significance. This analysis highlights specific metabolic pathways that are perturbed by the overexpression of *SLC25A20*. **C-L.** Relative abundance of selected differentially altered metabolites. These box plots show the relative abundance of specific metabolites in SLC25A20-overexpressing cells compared to control cells.

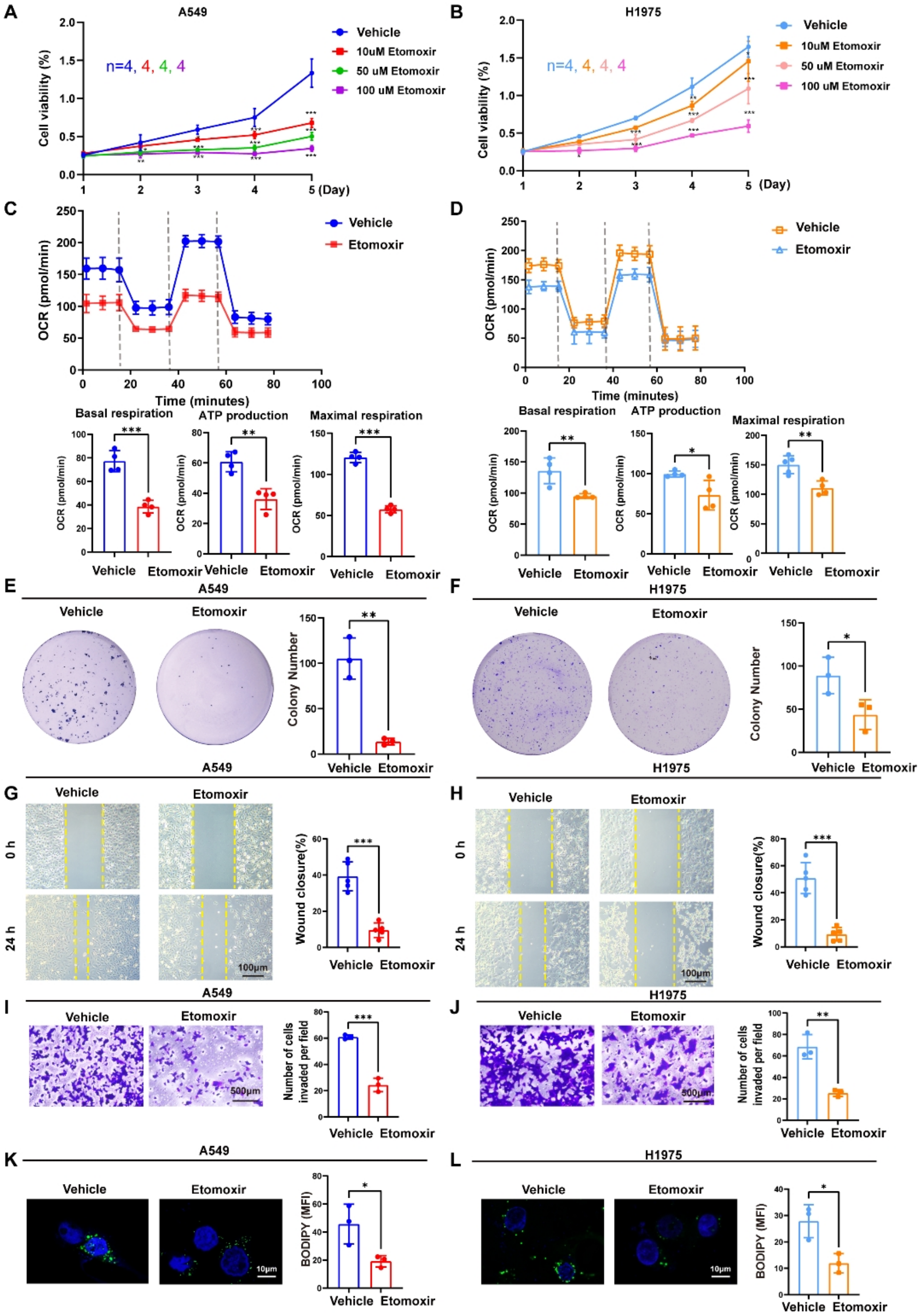
A
A549
Cell viability (%)
n=4, 4, 4, 4
Vehicle
10uM Etomoxir
50 uM Etomoxir
100 uM Etomoxir
(Day)
B
H1975
Cell viability (%)
n=4, 4, 4, 4
Vehicle
10uM Etomoxir
50 uM Etomoxir
100 uM Etomoxir
(Day)
C
OCR (pmol/min)
Time (minutes)
Vehicle
Etomoxir
Basal respiration
ATP production
Maximal respiration
OCR (pmol/min)
Vehicle
Etomoxir
D
OCR (pmol/min)
Time (minutes)
Vehicle
Etomoxir
Basal respiration
ATP production
Maximal respiration
OCR (pmol/min)
Vehicle
Etomoxir
E
A549
Vehicle
Etomoxir
Colony Number
F
H1975
Vehicle
Etomoxir
Colony Number
G
A549
Vehicle
Etomoxir
0 h
24 h
100μm
Wound closure(%)
H
H1975
Vehicle
Etomoxir
0 h
24 h
100μm
Wound closure(%)
I
A549
Vehicle
Etomoxir
500μm
Number of cells invaded per field
J
H1975
Vehicle
Etomoxir
500μm
Number of cells invaded per field
K
A549
Vehicle
Etomoxir
10μm
BODIPY (MFI)
L
H1975
Vehicle
Etomoxir
10μm
BODIPY (MFI)

Supplementary Figure 6. Etomoxir suppresses cell growth, mitochondrial respiration, invasion, and lipid accumulation in LUAD cells.

A, B. Cell viability of A549 and H1975 cells treated with vehicle or the indicated concentrations of Etomoxir at the indicated time points. **C, D.** Oxygen consumption rate (OCR) profiles of A549 and H1975 cells treated with vehicle or Etomoxir, as determined by Seahorse extracellular flux analysis. Quantification of basal respiration, ATP-linked respiration, and maximal respiration is shown below the OCR profiles. **E, F.** Colony formation assays of A549 and H1975 cells treated with vehicle or Etomoxir. Representative images and quantification of colony numbers are shown. **G, H.** Wound-healing assays of A549 and H1975 cells treated with vehicle or Etomoxir. Representative images at 0 and 24 h and quantification of wound closure are shown. **I, J.** Matrigel-coated Transwell invasion assays of A549 and H1975 cells treated with vehicle or Etomoxir. Representative images and quantification of invaded cells per field are shown. **K, L.** Representative BODIPY 493/503 staining images and quantification of neutral lipid accumulation in A549 and H1975 cells treated with vehicle or Etomoxir. Nuclei were counterstained with DAPI. Scale bars, 10 μm. Data are presented as mean ± SD from three independent experiments. Statistical significance was determined by two-tailed Student's t-test for two-group comparisons and two-way ANOVA for cell viability curves. $^*P < 0.05$, $^{**}P < 0.01$, $^{***}P < 0.001$.

Supplementary Table 1. Basic clinical information of samples in this study

No.	Patient ID	Age	Gender	Pathological stage	Clinical stage	T stage	N stage	M stage
1	1	59	F	Lung adenocarcinoma	Ⅰ A	1	0	0
2	2	54	F	Lung adenocarcinoma	Ⅰ A	1	0	0
3	3	56	F	Lung adenocarcinoma	Ⅰ B	2a	0	0
4	4	62	M	Lung adenocarcinoma	Ⅲ A	2	2	X
5	5	68	F	Lung adenocarcinoma	Ⅰ B	2	0	0
6	6	61	M	Lung adenocarcinoma	Ⅲ B	3	2	X
7	7	74	M	Lung adenocarcinoma	Ⅰ A	1	0	0
8	8	59	M	Lung adenocarcinoma	Ⅰ B	2	0	0
9	9	64	F	Lung adenocarcinoma	Ⅲ B	3	1	X
10	10	76	M	Lung adenocarcinoma	Ⅱ A	2	0	X
11	11	44	M	Lung adenocarcinoma	Ⅰ A	1	0	X
12	12	67	F	Lung adenocarcinoma	Ⅰ A	1	0	X
13	13	78	M	Lung adenocarcinoma	Ⅰ A	2	0	X
14	14	59	M	Lung adenocarcinoma	Ⅰ A	1	0	X
15	15	63	M	Lung adenocarcinoma	Ⅰ A	1	0	X
16	16	80	M	Lung adenocarcinoma	Ⅰ A	1mi	0	X
17	17	60	M	Lung adenocarcinoma	Ⅰ A	1	X	X
18	18	52	F	Lung adenocarcinoma	Ⅰ B	2	X	X
19	19	66	F	Lung adenocarcinoma	Ⅰ A	1mi	0	X
20	20	60	F	Lung adenocarcinoma	Ⅰ A	1	0	X
21	21	46	F	Lung adenocarcinoma	Ⅰ A	1	0	X
22	22	67	F	Lung adenocarcinoma	Ⅱ B	1	1	X
23	23	70	M	Lung adenocarcinoma	Ⅰ A	1	0	X
24	24	77	F	Lung adenocarcinoma	Ⅰ A	1	0	X
25	25	62	M	Lung adenocarcinoma	Ⅰ A	1mi	0	X
26	26	59	M	Lung adenocarcinoma	Ⅰ A	1	0	X
27	27	59	F	Lung adenocarcinoma	Ⅱ A	2	0	X
28	28	90	F	Lung adenocarcinoma	Ⅰ A	1	0	X

Supplementary Table 2. Functional annotation of representative metabolites identified by metabolomic analysis

Metabolite Name	Metabolite Ontology Class	Associated Metabolic Pathways	Metabolite Name	Metabolite Ontology Class	Associated Metabolic Pathways
NADPH	Nucleosides, nucleotides, and analogues	Lipid synthesis, Steroid biosynthesis, Oxidative stress defense	NADPH	Nucleosides, nucleotides, and analogues	Lipid synthesis, Steroid biosynthesis, Oxidative stress defense
Dephospho-CoA	Vitamins and derivatives	Fatty acid oxidation (FAO), Coenzyme A biosynthesis	Dephospho-CoA	Vitamins and derivatives	Fatty acid oxidation (FAO), Coenzyme A biosynthesis
Palmitoylcarnitine	Acyl carnitines	Fatty acid oxidation (FAO), Mitochondrial fatty acid transport	Palmitoylcarnitine	Acyl carnitines	Fatty acid oxidation (FAO), Mitochondrial fatty acid transport
L-Acetylcarnitine	Acyl carnitines	Fatty acid oxidation (FAO), Acetyl-CoA metabolism	L-Acetylcarnitine	Acyl carnitines	Fatty acid oxidation (FAO), Acetyl-CoA metabolism
Phytosphingosine	Sphingolipids	Sphingolipid metabolism, Lipid signaling	Phytosphingosine	Sphingolipids	Sphingolipid metabolism, Lipid signaling
9-Hexadecenoylcar-nitine	Acyl carnitines	Fatty acid oxidation (FAO)	10-Hexadecenoylcar-nitine	Acyl carnitines	Fatty acid oxidation (FAO)
Octanoylcarnitine	Acyl carnitines	Fatty acid oxidation (FAO)	Octanoylcarnitine	Acyl carnitines	Fatty acid oxidation (FAO)
3-Hydroxyhexanoyl-carnitine	Acyl carnitines	Fatty acid oxidation (FAO), β-oxidation	4-Hydroxyhexanoyl-carnitine	Acyl carnitines	Fatty acid oxidation (FAO), β-oxidation
Sphingosine 1-phosphate	Sphingolipids	Sphingolipid metabolism, Cell signaling	Sphingosine 2-phosphate	Sphingolipids	Sphingolipid metabolism, Cell signaling
Butyrylcarnitine	Acyl carnitines	Fatty acid oxidation (FAO)	Butyrylcarnitine	Acyl carnitines	Fatty acid oxidation (FAO)
Oleoylcarnitine	Acyl carnitines	Fatty acid oxidation (FAO)	Oleoylcarnitine	Acyl carnitines	Fatty acid oxidation (FAO)
5-Methyltetrahydrof-olic acid	Vitamins and derivatives	One-carbon metabolism, Nucleotide synthesis	6-Methyltetrahydrof-olic acid	Vitamins and derivatives	One-carbon metabolism, Nucleotide synthesis
Phosphoenolpyru-vic acid	Organic acids and derivatives	Glycolysis, Gluconeogenesis	Phosphoenolpyru-vic acid	Organic acids and derivatives	Glycolysis, Gluconeogenesis
S-Adenosylhomocy-steine	Amino acids, peptides, and analogues	Methionine metabolism, Methylation reactions	S-Adenosylhomocy-steine	Amino acids, peptides, and analogues	Methionine metabolism, Methylation reactions
L-Arginine	Amino acids, peptides, and analogues	Urea cycle, Nitric oxide synthesis	L-Arginine	Amino acids, peptides, and analogues	Urea cycle, Nitric oxide synthesis

Supplementary Table 3. qRT-PCR primer sequences.

qhFASN-F	AAGGACCTGTCTAGGTTTGATGC
qhFASN-R	TGGCTTCATAGGTGACTTCCA
qhFADS2-F	TGACCGCAAGGTTTACAACAT
qhFADS2-R	AGGCATCCGTTGCATCTTCTC
qhLDLR-F	GAGAGCTTGTGCCGAGATGTG
qhLDLR-R	CCGCAGTTGTTAGTGCCATCA
qhDHCR7-F	GCTGCAAAATCGCAACCCAA
qhDHCR7-R	GCTCGCCAGTGAAAACCAGT
qhSNAI2-F	CGAACTGGACACACATACAGTG
qhSNAI2-R	CTGAGGATCTCTGGTTGTGGT
qhSMAD6-F	CCTCCCTACTCTCGGCTGTC
qhSMAD6-R	GGTAGCCTCCGTTTCAGTGTA
qhID2-F	CGTTGCCCTATCTCCGTCTC
qhID2-R	GGGAGCAATCGGGTAATTTTCC
qhGAPDH-F	GGAGCGAGATCCCTCCAAAAT
qhGAPDH-R	GGCTGTTGTCATACTTCTCATGG

SupplementaryTable 4.sgRNA sequences used for SLC25A20 knockout.

sgRNA　Sequence (5′→3′)
sg1：ggcgtgtgcctggtgttcgt
sg2：cgtcggtcaccctctggaca
sg3：ggttgtccaggcaaactcgg
