# Supplementary material for: SLC25A20 promotes tumor growth by reprogramming energy metabolism in lung adenocarcinoma
Source: Cell Death Dis. 2026 Jul 23;17(1):893. doi: 10.1038/s41419-026-09119-9 (PMC13680618; doi:10.1038/s41419-026-09119-9)
Supplement: Supplementary file 3 [file 41419_2026_9119_MOESM1_ESM.docx]

Figure 1A

N1 T1 N2 T2 N3 T3

SLC25A20	GAPDH

N4 T4 N5 T5 N6 T6

SLC25A20	GAPDH

Figure 1E

**BESA-2B H1299 A549 H1975 H1650**

SLC25A20	GAPDH

Figure 2A

H1975

SLC25A20	GAPDH

H1650

SLC25A20	GAPDH

Figure 2H

H1975

Pro Caspase3	Caspase 3(cleaved)	β-actin

H1650

Pro Caspase3 Caspase 3(cleaved) β-actin

Figure 3A

A549

SLC25A20	β-actin

H1299

SLC25A20	β-actin

Figure 5H

FASN	GAPDH

HMOX1	GAPDH

Supplementary. Fig 3B

E-cadherin	Vimentin	GAPDH

Supplementary. Fig 4B

FASN	GAPDH

HMOX1	GAPDH
